# Interaction of Cytochrome P450 3A4 with Fatty Acid Binding Protein 1 and Relevance to Drug Metabolism

**DOI:** 10.1021/acs.jmedchem.5c03754

**Published:** 2026-03-04

**Authors:** Kevin D. McCarty, Destiny L. D. Proffett, F. Peter Guengerich

**Affiliations:** From the Department of Biochemistry, 5718 Vanderbilt University School of Medicine, Nashville, Tennessee 37232-0146, United States

## Abstract

Liver fatty acid binding protein (FABP1) is an abundant cytosolic component that interacts with drugs and can transfer substrates to cytochrome P450 enzymes (P450s, CYPs). FABP1 bound the P450 3A4 substrates diazepam and sulfinpyrazone with *K*_d_ values < 2.5 μM. FABP1 weakly attenuated P450 3A4 oxidations of both drugs in two independent assay modes. Reconstitution of human liver microsomes with cytosol attenuated diazepam metabolism but stimulated sulfinpyrazone oxidation, possibly due to GST A1-1. Kinetic modeling of reactions with varying FABP1 and substrate concentrations favored a model of directed substrate transfer to P450 3A4. Kinetic simulations revealed FABP1-dependent inhibition of diazepam oxidation at high physiological concentrations of FABP1, likely due to competition for P450 binding. Consideration of the influence of both P450 and FABP1 on drug metabolism may be critical for accurate modeling and prediction of drug pharmacokinetics.

## Graphical abstract

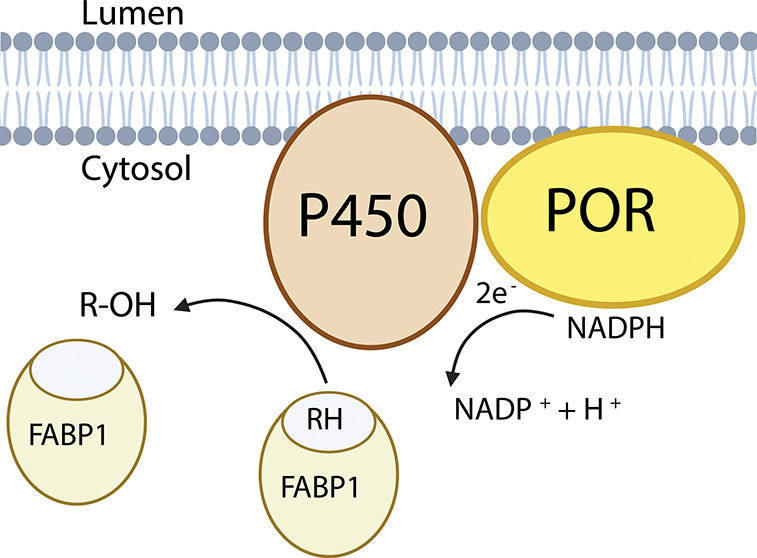

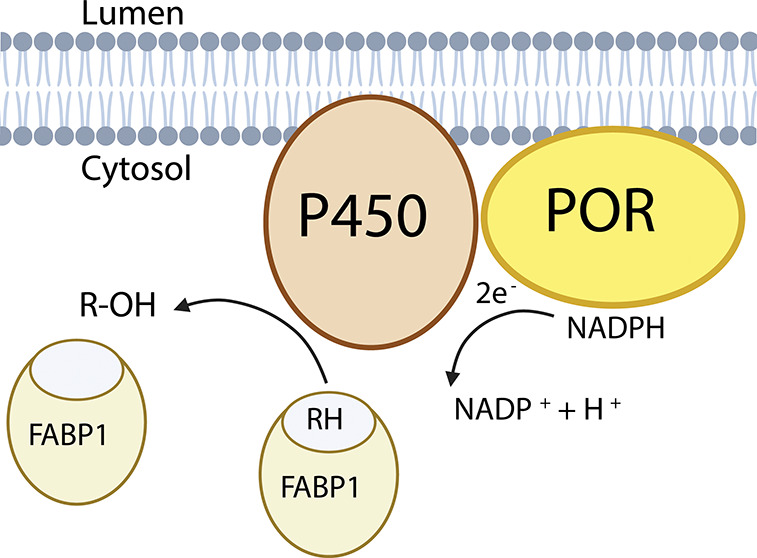

## Introduction

The accurate prediction of human hepatic drug clearance is crucial to the development of safe and efficacious small molecule therapeutics. Central to the process of physiologically based pharmacokinetic (PBPK) modeling is the extrapolation from experimentally determined in vitro drug metabolism parameters to estimate human intrinsic clearance in vivo, i.e., in vitro to in vivo extrapolation (IVIVE). IVIVE is crucial in the early optimization and selection of drug dosing and the prediction of drug–drug interactions, reducing–in theory–the experimental load and cost of early drug discovery. However, inconsistent measurements of in vitro parameters (e.g., between separate in vitro systems) can preclude accurate IVIVE of the corresponding drugs and present a logistical challenge to discern the experimental basis of the discrepancy. Contrasting in vitro results derived from differences in system composition (such as the presence and contribution of certain biological factors^1,2^ ) can raise important questions in addressing IVIVE and PBPK modeling.

Reactions catalyzed by cytochrome P450 (P450, CYP) 3A4 present interesting and sometimes challenging cases for PBPK modeling. P450 3A4 dominates hepatic drug metabolism, constituting ∼30% of the total P450 content of human liver^3^ (though this figure can be as high as 60% in some individuals^4^) and is responsible for the metabolism of up to ∼50% of all pharmaceutical drugs.^5,6^ A reported difficulty of IVIVE of some P450 3A4 reactions has been a persistent issue in recapitulating drug metabolism parameters assayed in liver microsomes with results from human hepatocytes. Termed the “CYP3A4 anomaly,^7^” the phenomenon describes a reproducible and differential effect of the selection of in vitro system on drug metabolism kinetics. Reportedly, a majority of P450 3A4 substrates display higher in vitro clearance (i.e., *k*_cat_/*K*_m_) in assays with liver microsomes than with intact hepatocytes–an effect that has not been observed in the reactions of some other P450 enzymes.^2,7,8^ Explanations for this effect are variable and have included possible coordinated regulation of the efflux transporter *P*-glycoprotein, low passive permeability, and limited cofactor availability, though these explanations^2^ (and the existence of the effect itself^9^) have been challenged.

The consequences of anomalous P450 3A4 kinetic measurements in the early stages of drug discovery can be significant, given that liver microsomes are a primary screening system for metabolic stability in the vetting of candidate molecules.^10^ Assays using microsomes may yield anomalously high clearance for a particular drug candidate, but this may not be inherently obvious unless the corresponding assay is also performed in hepatocytes (i.e., to reveal the discrepancy). Hepatocyte systems are more expensive (in comparison to liver microsomes) and their use can require additional considerations (e.g., regarding passive permeability and efflux of candidate drugs), thus providing a less convenient and cost-effective option for large-scale early stage screening assays.^11^ It is worth noting that permeabilized hepatocytes have been used to mitigate these limitations (advantageous for permeability-limited drugs), though these systems often leak cofactors and cytosolic proteins^12^ and are also costly. Artifactual overestimation of microsomal clearance can result in the premature termination of candidate drugs that may–in actuality–be viable prospects, potentially culminating in missed opportunities for pharmaceutical companies and unrealized therapeutics for patient populations. The identification of contributing factors to the CYP3A4 anomaly may offer strategies to mitigate anomalous data and improve IVIVE of P450 3A4 reactions in drug discovery efforts.

One obvious difference between the two in vitro approaches is system composition: i.e., the presence (hepatocytes) or absence (microsomes) of cellular cytosol. Intriguingly, some reactions in xenobiotic metabolism have been reported to be effected by cytosol, e.g., stimulation of the metabolism of the carcinogen *N*,*N*-dimethyl-4-aminoazobenzene by liver cytosol.^13^ Fatty acid binding protein 1 (FABP1) constitutes a significant portion (∼7–11%, up to 1 mM intracellular concentration) of liver cytosolic protein^14^ and serves a critical function as an intracellular lipid chaperone, binding and targeting fatty acids to organelles (as well as model membranes^15,16^ ) bearing fatty acid oxidation machinery (i.e., mitochondria, peroxisomes, endoplasmic reticulum).^14^ FABPs are also reported to interact with nuclear receptors (e.g., PPARs^17,18^ ), and our laboratory has further demonstrated that FABP1 transfers fatty acid substrates to P450 4A11 (a fatty acid ω-hydroxylase) for metabolism via a direct protein–protein interaction.^19^ However, as has been recently reviewed by the Isoherranen group, the ligand pool of FABPs is not limited to fatty acids, and a rather broad role for FABPs in the binding of hydrophobic molecules (i.e., other lipids, steroids, cannabinoids, and some pharmaceutical drugs) has been invoked–including a number of substrates of P450 3A4.^20^

FABP1 has been implicated as an effector of hepatic drug metabolism in recent studies by the Isoherranen group, which described the FABP1-mediated inhibition of the metabolism of pharmaceutical drugs by P450 2C9 ^21^ and Δ^9^-cannabinoids by P450s 2C9, 2C19, and 3A4.^22^ Building upon this, their group has recently reported the development of a physiologically based liver distribution model that offers substantially improved distribution prediction when a parameter for intracellular drug-binding to FABP1 was included.^23^ Their simulations revealed that high physiological concentrations of FABP1 (estimated at ∼400 μM via their quantitative proteomics approach with 61 human livers) effect hepatic drug concentrations even when drug-binding to FABP1 is relatively weak (*K*_d_ value ∼5 to 20 μM).

Another abundant component of liver cytosol is glutathione transferase A1-1 (GST A1-1), representing roughly ∼2 to 5% of the total protein content.^24,25^ First identified for its properties as a “ligandin” (i.e., binding steroids, drugs, and carcinogens^26^), the role of GST A1-1 in drug conjugation has been well established: the “partnering” of GST reactions with P450 reactions is known, in that the enzymes can be sequential partners in the oxidative (so-called “Phase I”) and conjugative (“Phase II”) metabolism of drugs.^27^ Metabolic products generated in Phase I by P450 enzymes can be conjugated to glutathione by GST A1-1 in Phase II for excretion. However, whether competition of P450s and GST A1-1 for substrates or direct interaction of the two proteins occurs is less clear.

Given (i) that intrinsic clearance of P450 3A4 substrates is frequently overpredicted by liver microsomes; (ii) that FABP1 is an abundant component of hepatic cytosol that is absent in microsomes; (iii) that FABP1 binds pharmaceutical drugs (including P450 3A substrates) and reportedly alters drug metabolism; and, (iv) that accounting for drug-binding by FABP1 is important for the accurate prediction of tissue drug distribution, we questioned whether the binding of P450 3A4 substrates to FABP1 alters their metabolism such that an increased microsomal clearance (relative to hepatocytes, i.e., the “CYP3A4 anomaly”) may be partially explained by the presence or absence of cytosolic proteins in the in vitro assays. We postulated that FABP1 represents a cytosolic effector of P450 3A4 reactions that is generally unaccounted for in PBPK models and that its absence complicates the accurate determination of in vitro kinetic parameters. While current PBPK modeling accounts for the effect of abundant extracellular binding proteins (e.g., serum albumin) on drug distribution and metabolism (i.e., via sequestering P450 substrates), the potential influence of intracellular binding proteins has been largely overlooked^23^ (although some have reported that the addition of dense extracellular protein (e.g., serum, plasma,^28^ or BSA^1^) to microsomal experiments improves IVIVE–potentially explained by molecular crowding^29^–though others have failed to reproduce the effect).^30^

In this study, we employed both recombinant proteins and liver microsomes to assess the kinetic impact of FABP1 and GST A1-1 on P450 3A4 reactions catalyzed with drugs that bound tightly to FABP1. We screened 13 P450 3A4 substrates for FABP1-binding, identifying sulfinpyrazone and diazepam with relatively low (<2.5 μM) *K*_d_ values, and measured binding constants and kinetics of both drugs and their metabolites to P450 3A4 and FABP1. We tested the effects of recombinant FABP1 and GST A1-1 on P450 3A4 incubations with both drugs and attempted to recapitulate those results in liver microsomes reconstituted with cytosol. Finally, we measured the FABP1-P450 3A4 association kinetics using a fluorophore-conjugated FABP1 and assembled a kinetic model of the FABP1-P450 3A4 interaction. From the results, we developed a kinetic model of the interaction and conclude that FABP1 can directly transfer pharmaceutical substrates to P450 3A4.

## Results and Discussion

### Rationale

FABP1 is reportedly expressed at intracellular concentrations of ≥400 μM,^14,23^ a value that greatly outnumbers the microsomal expression of P450 enzymes in liver cells (at least ∼400-fold, based on rough estimates of typical microsomal P450 content^4^) (Figure 1). The capacity of FABP1 to bind P450 substrates (fatty acids, steroids, drugs) has been reported,^20^ with our own group characterizing FABP1 as a substrate chaperone to a P450 fatty acid oxidase^19^ and another group reporting FABP1-dependent inhibition of the metabolism of some drugs.^21,22^ Whether FABP1 acts in competition (competition model, free drug hypothesis) or in complementation (direct transfer model) of hepatic drug metabolism by P450 3A4 is the fundamental question of the present work (Scheme 1).

**Figure 1:**
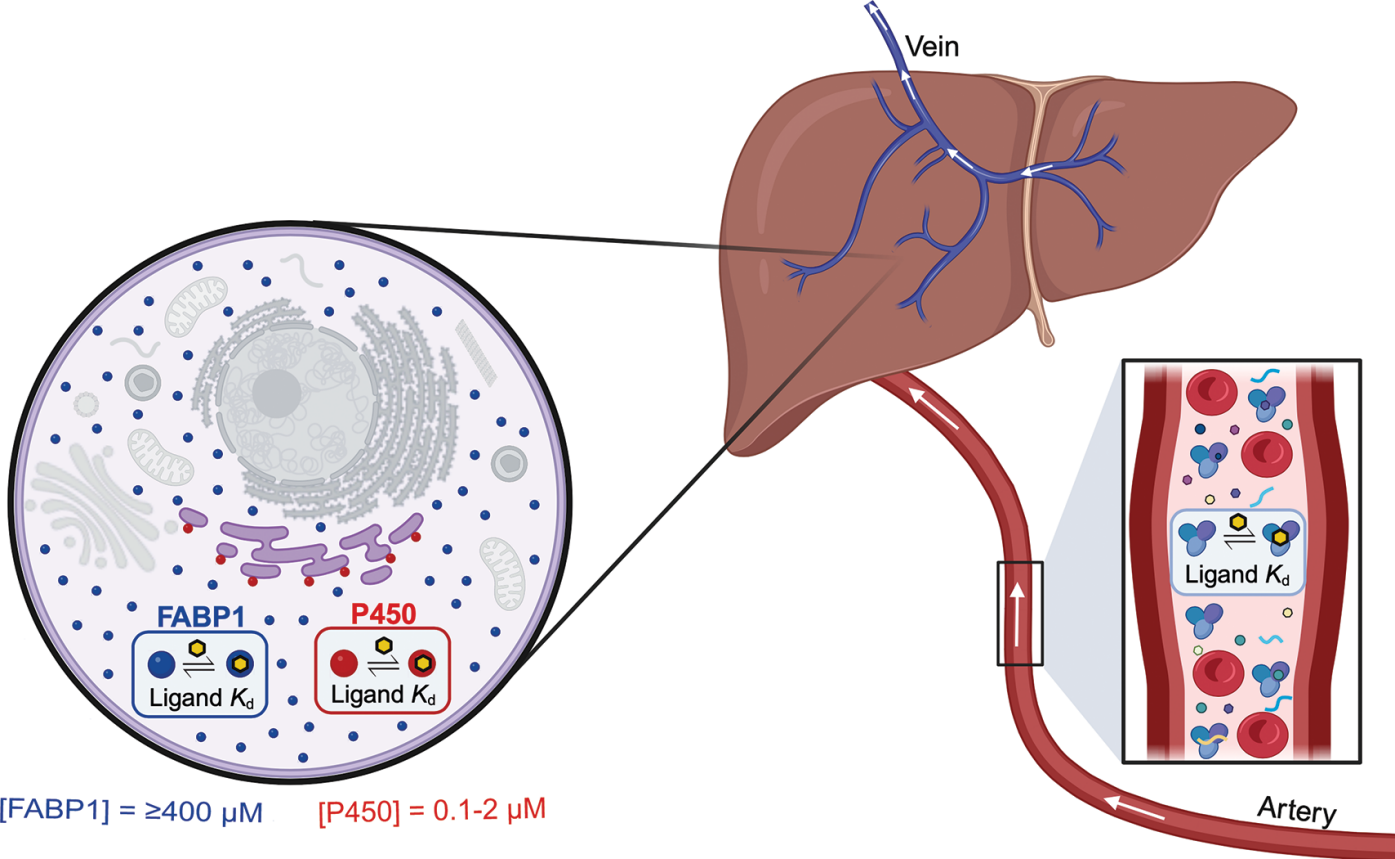
Protein-binding of small molecules and the impact on drug metabolism. The role of the extracellular binding proteins (inset box) in complexing P450 substrates (yellow hexagon) in the serum and delaying their metabolism is well-characterized. The “fraction unbound (*f*_u_)” of a drug refers to the percent of the drug dose that is not bound by serum proteins and is a critical parameter in predicting drug pharmacokinetics. Once a drug molecule enters the hepatocyte (inset circle), whether intracellular ligandins such as FABP1 (blue circle) bind and delay P450 (red circle) metabolism, constituting an intracellular *f*_u_, is the question of the present work. Similar to serum albumin, FABP1 is highly expressed in comparison to P450 enzymes. The direction of blood flow into (via arteries) and out of (via veins) the liver is indicated with arrows. Nonparticipatory organelles are colored in gray. Image made with BioRender.com.

**Scheme 1:**
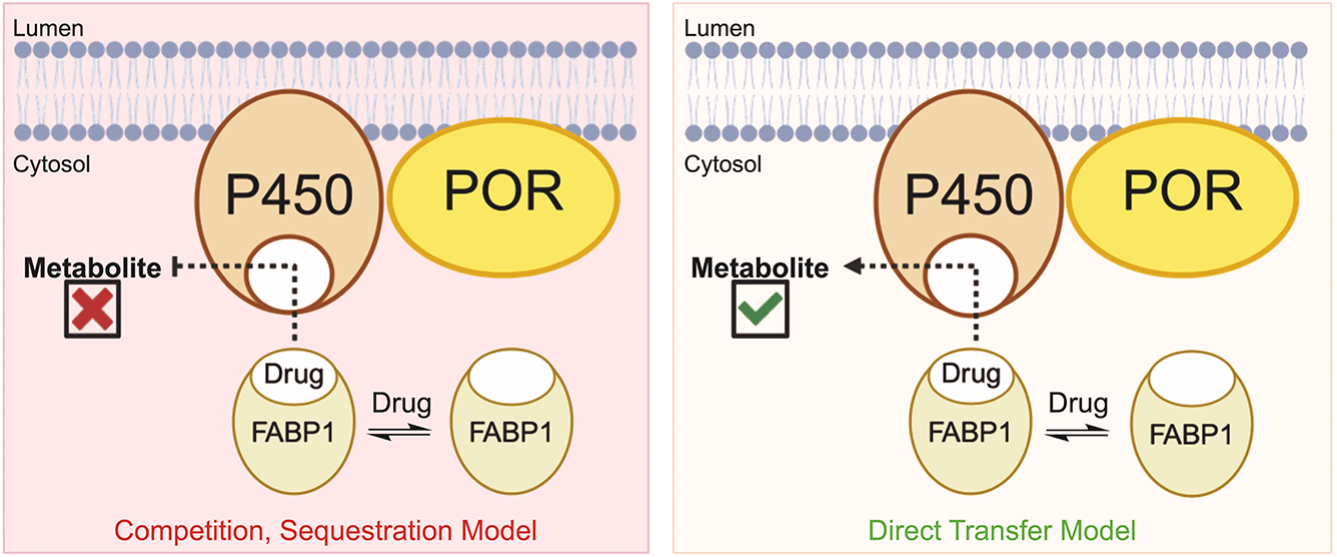
A Simplified Model of FABP1 in Drug Metabolism^a^

a In a competition model (left, shaded in red), FABP1 (green) binds and sequesters the P450 substrate (“drug”) from metabolism (No metabolite formation). The binding of drugs by FABP1 is inhibitory to P450 (red) metabolism. In a direct transfer model (right, shaded in green), FABP1 binds a drug molecule and transfers it to P450 for metabolism, yielding a drug metabolite. Image made with BioRender.com.

Herein, we screened 13 P450 3A4 substrates for tight FABP1-binding and revealed the influence of FABP1 on the P450 3A4-mediated oxidation of two drugs following a kinetic characterization (Scheme 2) and computational modeling of: (i) rate and binding constants of both drugs to each protein; (ii) P450 3A4-FABP1 interaction kinetics; (iii) the effect of FABP1 on P450 3A4 drug oxidation kinetics; and, (iv) the effect of (FABP1-rich) human liver cytosol on microsomal incubations with both drugs. The impact of the ligandin glutathione transferase GST A1-1 on both reactions was also considered. In the cases in which it was not possible to measure a kinetic parameter, it was estimated via computational modeling (unless otherwise noted). The experimentally informed computation model was then used to extrapolate to cellular concentrations of FABP1, GST A1-1, and P450 3A4 to predict the effects of protein-binding on P450 3A4 oxidations in hepatocytes.

**Scheme 2:**
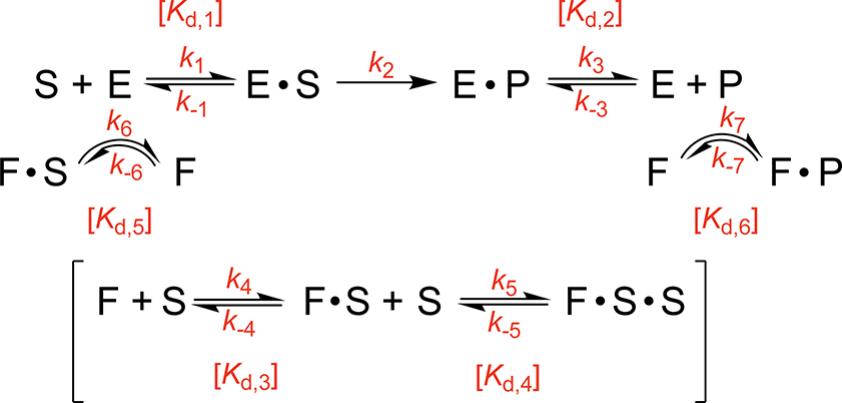
A Kinetic Model of FABP1 in Drug Metabolism^a^

a P450 (E) binds a drug molecule (S) and oxidizes it to a product (P). FABP1 (F) binds S to form a complex (FS) that can accommodate up to two molecules of substrate (i.e., FSS, shown in brackets). All dissociation constants and rate constants are labeled in red. The value of rate constant *k*_6_ is a critical distinguishing factor between the “competition” and “direct transfer” models (Scheme 1): a competition model predicts that E only binds free S and that E and F do not interact (i.e., *k*_6_ = 0) while the direct transfer model conversely that E and F interact (i.e., *k*_6_ = nonzero).

### FABP1 Drug-Binding Screen

To screen drug-binding to FABP1, we heterologously expressed and purified recombinant FABP1 as previously described.^19^ We carefully removed endogenous bacterial fatty acids from the protein solution (scavenged during recombinant expression in *Escherichia coli*) that would interfere with a sensitive drug-binding assay using a hydrophobic Sepharose resin (Lipidex-5000). Delipidation was verified via native mass spectrometry, and the protein was quantified by both *A*_280_ (ε_280_ = 1490 M^–1^ cm^–1^) and by free thiol quantitation (using Ellman’s reagent, DTNB) to ensure an accurate measurement (values were within ∼6% of each other, data not shown). The characterization of purity and quality of relevant chemicals and proteins (LC–MS, SDS-PAGE) are provided in the Supporting Information (Table S1, Figures S1–S10).

Various approaches have been employed to assess the binding of molecules to FABP1, including isothermal titration calorimetry, surface plasmon resonance, and competition assays.^20,31–33^ Perhaps most commonly employed however are fluorophore displacement assays, wherein the binding of a fluorophore to FABP1 stimulates fluorescence that is attenuated when the fluorophore is displaced by the binding of another molecule. The effect is displayed in Figure 2 and can be used to estimate dissociation constants. The most common fluorophores used with FABP1 have been 8-anilinonapthalenesulfonic acid (ANS) and 11-dansylaminoundecanoic acid (DAUDA).^34,35^

**Figure 2:**
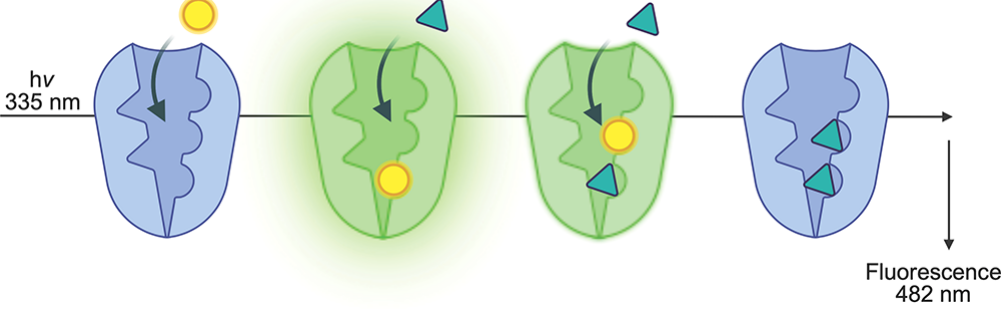
Methodology of the FABP1 drug-binding screen.^34^ When a molecule of DAUDA fluorophore (yellow circle) enters the FABP1 (blue) binding cavity, a significant gain of fluorescence (at 482 nm) is observed (represented by green luminescence of the FABP1-DAUDA complex). When a drug molecule (teal triangle) binds to the DAUDA-FABP1 complex (green), DAUDA is displaced (to a second binding site, or the FABP1-DAUDA complex is altered), and the resulting ternary complex is markedly less fluorescent (shown as diminished green luminescence). The displacement of DAUDA by a second molecule of substrate obliterates the fluorescence. Image made with BioRender.com.

Both fluorophores were initial candidates for our fluorescence screening assay. Our previous experience with DAUDA had revealed a *K*_d_ value of 0.056 (±0.023) μM for FABP1 ^19^ while a value of 1.0 (±0.1) μM was measured for ANS, both of which were tighter than literature estimates of 0.4 to 1.4 μM and 1.1 to 6 μM, respectively.^21,36^ Both systems were then calibrated with palmitic acid binding and compared to literature values (38 (±1) nM),^37^ as palmitate is a physiological FABP1 substrate. The value observed with DAUDA-FABP1 was 0.07 μM but was 110 μM with ANS-FABP1. We then compared progesterone binding, where a literature estimate is 30 nM,^20^ and determined values of 13 (±2) μM with DAUDA-FABP1 and 1.0 (±0.1) μM with ANS-FABP1 (original spectra and binding plots for all tested chemicals are presented in the Supporting Information, Figures S11–S14).

One complication with the ANS-FABP1 assay is the use of significant fluorophore excess (70 μM) relative to the protein concentration (0.5 μM).^38^ While we had reduced the ANS concentration (to 10 μM) for our assays, a significant amount of unbound fluorophore is still expected in the system. In comparison, the DAUDA-FABP1 assay utilized an excess of FABP1 (0.5 μM) over DAUDA (0.25 μM) (as is recommended for fluorescence assays^39^). Also, ANS is not a specific fluorophore for FABP1 and has been historically used as a general tool to identify hydrophobic cavities in proteins.^40^ Due to the lack of agreement between the two approaches, the noted caveats with the use of ANS, and the fact that DAUDA is a tightly bound and physiological substrate derivative of FABP1, the DAUDA-FABP1 system was selected for our fluorescence screen.

We screened 13 known substrates of P450 3A4 for binding to FABP1 (displacement of FABP1-DAUDA, Figure 2), considering structurally diverse molecules including steroids, carcinogens, and drugs (Table 1). Of those molecules, only three drugs bound tightly to FABP1: diazepam (*K*_d_ 2.3 (±0.7) μM), loratadine (*K*_d_ 1.2 (±0.1) μM), and sulfinpyrazone (*K*_d_ 1.7 (±0.2) μM) (Figures 3, S11–13). Of these, diazepam and sulfinpyrazone were selected for initial testing (Scheme 3). For the sake of comparison, we also performed titrations of diazepam, sulfinpyrazone, and temazepam with ANS-FABP1, measuring *K*_d_ values of 22 (±9) μM, 2.8 (±0.2) μM, and >50 μM, respectively, which are similar (within ∼2-fold) to the DAUDA-FABP1 values except in the case of diazepam, which is ∼10-fold weaker with ANS-FABP1.

**Table 1:** Comparison of Experimentally Determined and Literature Values for the Binding of 13 P450 3A4 Substrates to FABP1^b^

	descriptor	FABP1 *K*_d_ value, μM	
P450 3A4 substrate		experimental value	literature value
2-acetylaminofluorene	carcinogen	ND ≤ 50	NA
benzo[*a*]pyrene	carcinogen	ND ≤ 40	NA
codeine	drug	ND ≤ 60	NA
diazepam	drug	2.3 ± 0.7	0.5
loratadine	drug	1.2 ± 0.1	NA
midazolam	drug	21 ± 3	7.9
morphine	drug	ND ≤ 60	NA
*N*,*N*-dimethyl-4-aminoazobenzene	carcinogen	5.4 ± 1.5	NA
nifedipine	drug	36 ± 13	NA
progesterone	steroid	13 ± 2	0.03
sulfinpyrazone	drug	1.7 ± 0.2	0.1
temazepam	metabolite	35 ± 13	NA
testosterone	steroid	21 ± 2	NA
*R*-warfarin^a^	drug	5.8 ± 1.2	NA

a Although not a P450 3A4 substrate, the binding of *S*-warfarin (P450 subfamily 2C substrate) was also measured and a *K*_d_ value of 5.4 (±0.9) μM was estimated.

b Literature values from a FABP1 and drugs. ND ≤ X: binding not detected or saturable below the maximum concentration tested (X); NA, literature value unavailable.

**Figure 3:**
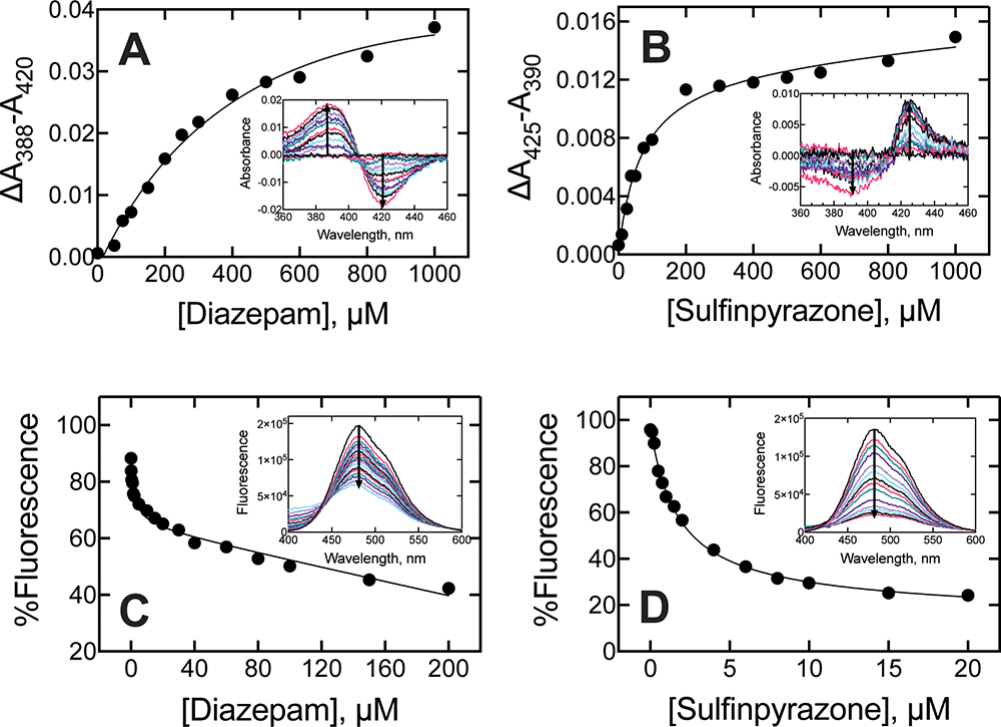
Binding constants (*K*_d_) of diazepam and sulfinpyrazone with P450 3A4 and FABP1. P450 3A4 (1 μM) was titrated with ethanolic solutions of (A) diazepam and (B) sulfinpyrazone. A blue-shifted (420 to 390 nm) heme Soret spectrum was observed with diazepam (A, inset), while a slightly red-shifted spectrum was observed with sulfinpyrazone (B, inset). Diazepam was not soluble at concentrations ≥1 mM. A complex of FABP1 (0.5 μM) and DAUDA (0.25 μM) was titrated with ethanolic solutions of (C) diazepam and (D) sulfinpyrazone and fluorescence emission (482 nm) was recorded and normalized to the starting fluorescence values. The binding of both drugs induced fluorescence attenuation (C and D, inset). All experiments were fit to single hyperbolic equations.

**Scheme 3:**
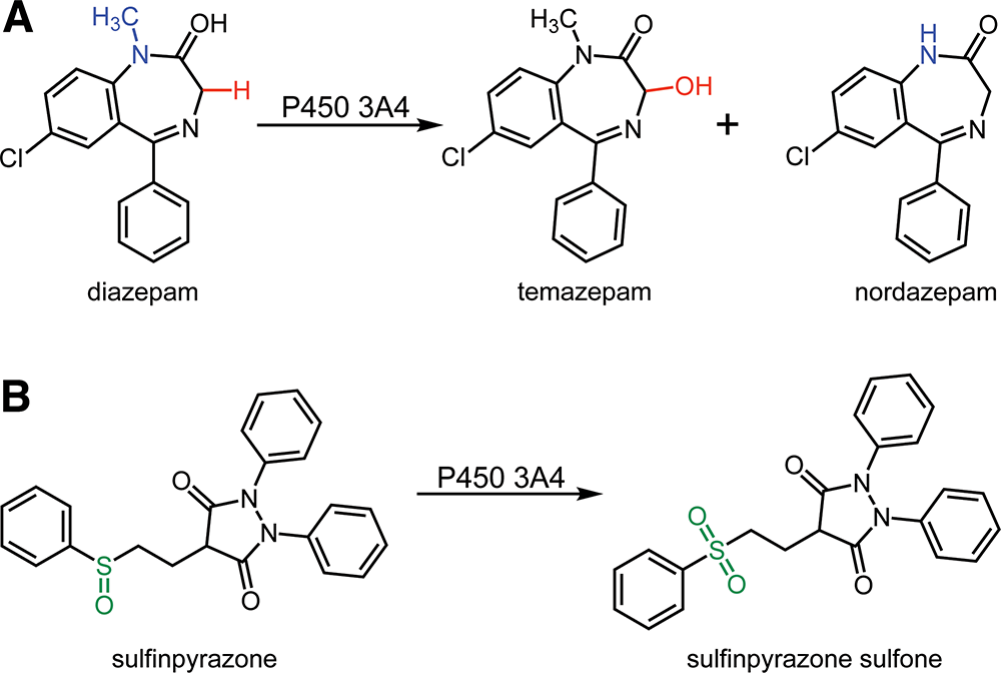
Reactions of P450 3A4 with Diazepam and Sulfinpyrazone^a^

a (A) P450 3A4 oxidizes diazepam to multiple products. The hydroxylation reaction (yielding temazepam) is the dominant reaction over the competing *N*-demethylation reaction (ratio of ∼3:1) yielding nordazepam. The oxidation sites are indicated in red and blue, respectively. (B) P450 3A4 oxidizes sulfinpyrazone (a sulfoxide) to a sulfone. The oxidation site is shown in green.

### Binding of Diazepam and Sulfinpyrazone to P450 3A4

The binding constants for sulfinpyrazone and diazepam were then assessed with recombinant P450 3A4 by monitoring the UV–visible spectral shift in the heme Soret band as drugs bind.^41,42^ For diazepam, the Soret was blue-shifted (420 to 390 nm) representing a classical “Type I” P450 substrate binding spectrum and yielding a *K*_d_ value of 620 (±380) μM (Figure 3, Table 2). Sulfinpyrazone binding to P450 3A4 was red-shifted (to 425 nm), appearing similar to a reverse-Type I (or possibly a (red-shifted) Type II) binding spectrum (Figure 3) and yielding a *K*_d_ value of 70 (±17) μM (Figure 3, Table 2). While rather unusual, we (and others) have previously reported red-shifted Soret spectra with the binding of some substrates and products to P450 enzymes.^19,43,44^ The variable but generally weak binding of drugs to P450 3A4 was unsurprising.

**Table 2:** Rates and Binding Constants of Drugs to Recombinant Proteins^a^

parameter	protein	diazepam	sulfinpyrazone	temazepam
*K*_d_, μM	FABP1	2.3 ± 0.7	1.7 ± 0.2	35 ± 13
	P450 3A4	620 ± 380	70 ± 17	>100
	GST A1-1	260 ± 130	97 ± 14	-
*k*_on_, (×10^6^ M^–1^ s^–1^)	FABP1	0.34 ± 0.01	0.27 ± 0.01	-
	P450 3A4	0.23 ± 0.006	0.024 ± 0.005	-
*k*_off_, s^–1^	FABP1	0.38 ± 0.02	0.267 ± 0.002	-
	P450 3A4	143	1.7	-

a Italicized parameters were estimated (solved for) from two known experimental values.

### Drug Binding Kinetics to P450 3A4 and FABP1

To assess the potential competition of FABP1 and P450 3A4 for substrate binding, measurement and comparison of drug binding kinetics was performed with both proteins (Scheme 2) using a stopped-flow apparatus. The binding of drugs to P450 3A4 was monitored spectrally (as previously), wherein solutions containing P450 solution (2 μM) and drug solution (100 μM) were rapidly mixed and the corresponding Soret spectral changes (Figure 3) were monitored as a function of time (Figure 4). The binding of both drugs to P450 3A4 was biphasic, which was unsurprising considering our previous work with the enzyme.^42^ Drug binding rates (*k*_on_) of the fast phases of diazepam and sulfinpyrazone binding to P450 3A4 were 2.3 (±0.1) × 10^5^ M^–1^ s^–1^ and 2.4 (±0.5) × 10^4^ M^–1^ s^–1^, respectively (Figure 4, Table 2).

**Figure 4:**
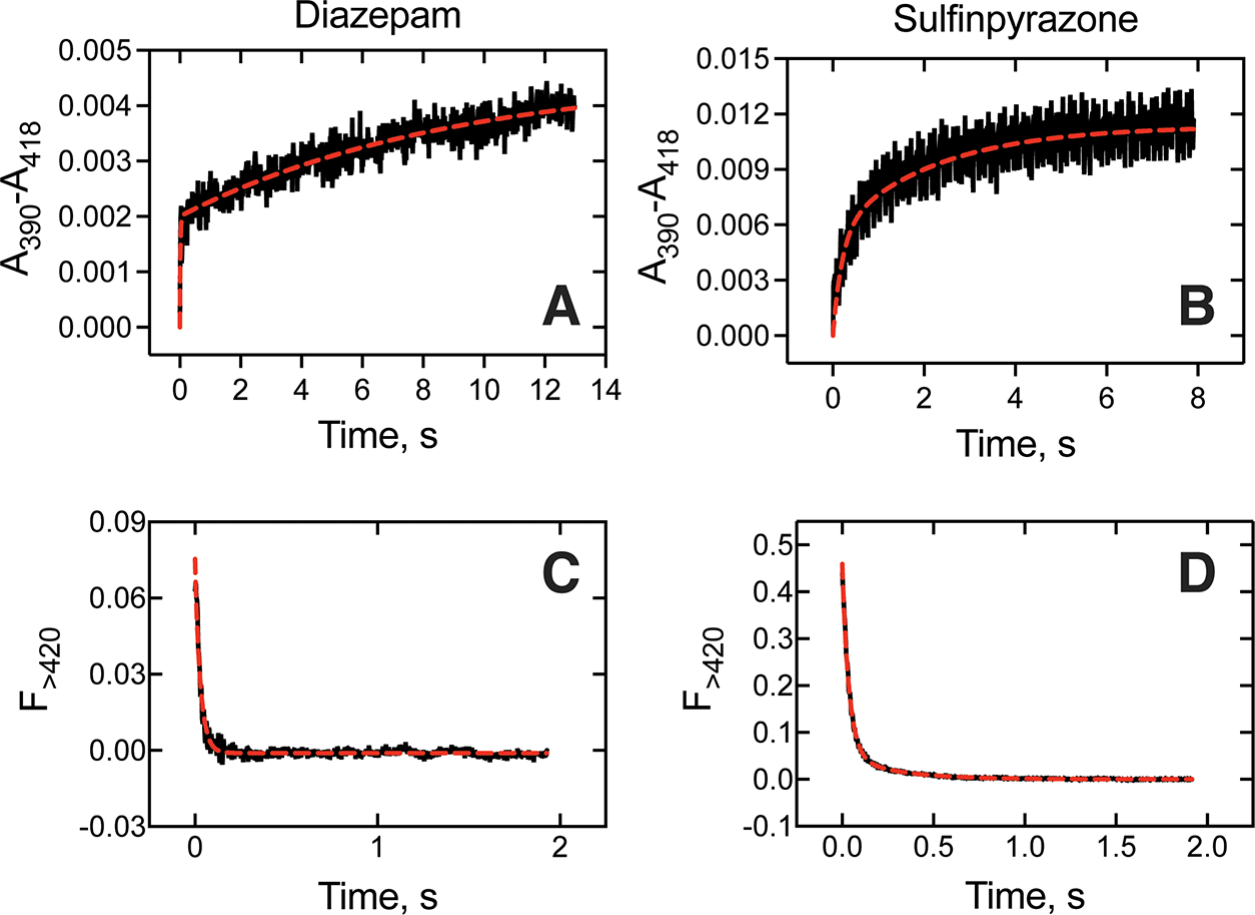
Binding rates (*k*_on_) of diazepam and sulfinpyrazone to P450 3A4 and FABP1. P450 3A4 (1 μM) was rapidly mixed with 100 μM solutions of (A) diazepam and (B) sulfinpyrazone prepared in potassium phosphate buffer (100 mM, pH 7.4). A blue-shifted (420 to 390 nm) heme Soret spectrum developed after mixing and each substrate bound to P450 3A4. The *k*_on_ rates of the fast phases of diazepam and sulfinpyrazone binding to P450 3A4 were 0.23 ± 0.01 × 10^6^ M^–1^ s^–1^ and 0.024 ± 0.005 × 10^6^ M^–1^ s^–1^, respectively. A complex of FABP1 (1 μM) and DAUDA (1 μM) was mixed against 100 μM solutions of (C) diazepam and (D) sulfinpyrazone and the total fluorescence output (≥420 nm) of the admixture was recorded. The *k*_on_ rates of diazepam and the fast phase of sulfinpyrazone binding to FABP1 were 0.34 (±0.01) × 10^6^ M^–1^ s^–1^ and 0.27 (±0.01) × 10^6^ M^–1^ s^–1^, respectively. Curve fits to exponential equations are represented as dashed red lines (all double exponentials except C). Each trace represents the average of ≥5 individual experiments.

Drug binding to FABP1 was estimated from displacement of the DAUDA fluorophore from DAUDA-FABP1 (as in the binding titrations, Figures 2 and 3) over time using a stopped-flow spectrofluorometer. The fluorescence attenuation observed with the binding of sulfinpyrazone to FABP1 was biphasic, which was unsurprising given our previous experience with FABP1.^19^ Diazepam binding was weaker in intensity and was fit well to a single exponential equation, suggesting less complexity in the binding process. Drug binding rates (*k*_on_) of diazepam and the fast phase of sulfinpyrazone binding to FABP1 were 3.4 (±0.1) × 10^6^ M^–1^ s^–1^ and 2.7 (±0.1) × 10^5^ M^–1^ s^–1^, respectively (Figure 4, Table 2). These rates are slower than what we have previously estimated for the binding of natural substrates (fatty acids) and the fatty acid derivative, DAUDA (9.0 × 10^7^ M^–1^ s^–1^).^19^

### Binding of P450 3A4 to FABP1

The ability of P450 3A4 to interact with FABP1 was assessed as previously described,^19^ using an Alexa Fluor-488 (cysteine-reactive) fluorophore-conjugated FABP1 derivative (Alexa-FABP1, labeled at the Cys-69 position of the WT protein). The binding of Alexa-FABP1 (25 nM) was assessed to human P450 3A4 in fluorescence titrations and compared to titrations with a bacterial P450 (P450_cam_, from *Pseudomonas putida*) and the bovine serum albumin (BSA). All protein titrations were normalized to the dilution of signal predictably observed in the buffer titration (the buffer titration also normalized the data for any fluorescence bleaching that may occur during the experiment).

Fluorescence stimulation (monitored at λ_emission_ of 510 nm) of Alexa-FABP1 by P450 3A4 was substantial and revealed a *K*_d_ value of 15.3 (±5.7) nM for the interaction (Figure 5). Stimulation by BSA was weaker but appeared saturable and yielded a *K*_d_ value (with a high margin of error) of 53 (±94) nM. Fluorescence stimulation by P450_cam_ was even weaker and not saturable in the tested concentration range (as observed previously).^19^

**Figure 5:**
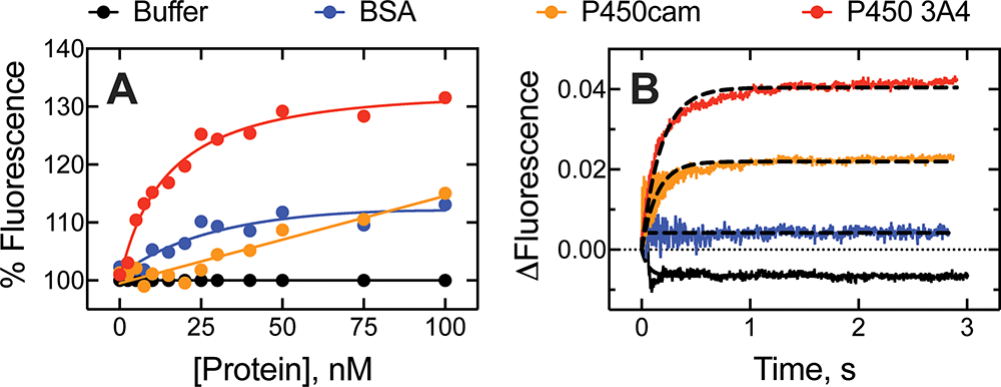
Binding of P450 3A4 to Alexa-FABP1. (A) Equilibrium binding titrations. Solutions of human P450 3A4 (red), P450_cam_ (orange), BSA (blue), and buffer (black) were titrated into a solution of FABP1 prepared in potassium phosphate buffer (100 mM, pH 7.4). Fluorescence emission values (recorded at 510 nm) were normalized to those observed in the buffer titration. Solid lines represent data fits to hyperbolae. (B) Estimation of P450 3A4-FABP1 *k*_on_ rate via stopped-flow fluorescence spectroscopy. The same proteins (0.25 μM) as in (A) were rapidly mixed with Alexa-FABP1 (0.25 μM) and the total fluorescence output (≥495 nm) of the admixture (0.125 μM) was recorded. Curve fits to single exponential equations are represented as dashed black lines. Each trace represents the average of ≥5 individual experiments.

The kinetics of P450-FABP1 binding were then evaluated with a stopped-flow spectrofluorometer, collecting total fluorescence output (≥495 nm) over time.^19^ The change in fluorescence is reported after normalization to the starting signal at the time of mixing, and ≥5 experiments (traces) were averaged to generate the data shown in Figure 5. Stimulation of Alexa-FABP1 (0.125 μM) fluorescence was most prominent and rapid with P450 3A4 (5.6 s^–1^), followed by P450_cam_ (7.1 s^–1^), revealing binding rates of 4.5 × 10^7^ M^–1^ s^–1^ and 5.7 × 10^7^ M^–1^ s^–1^ (assessed at final protein concentrations of 0.125 μM). These rates compare to our value of 2.1 × 10^7^ M^–1^ s^–1^ previously reported for the P450 4A11-Alexa-FABP1 interaction,^19^ and represent *k*_on_ rates within a reasonable range to be expected for a protein–protein interaction. Fluorescence stimulation by BSA was weak and near baseline, and control experiments with buffer alone only diluted the Alexa-FABP1 signal. The more significant stimulation by P450_cam_ (relative to BSA) observed in the rapid mixing experiment in comparison to the binding assay is likely due to the higher concentrations of protein used for stopped-flow measurements (0.125 μM). The fluorescence stimulation by P450_cam_ did not saturate in the binding assay and is expected to further increase with additional enzyme, while the BSA signal had reached a maximum (saturation) at ∼50 nM protein.

### Kinetic Modeling of Steady-State Incubations

To complement the in vitro studies, the effect of FABP1 on steady-state P450 3A4 reactions was kinetically modeled based on the predictions of the “competition model” and the “direct transfer model” described earlier (Scheme 1). The kinetic model was constructed as shown in Scheme 2 and was informed with kinetic parameters that were experimentally determined or estimated herein (where possible) and compiled in Table S2. The prediction of the competition model (i.e., that FABP1 and P450 3A4 compete for substrate binding and do not interact) was generated by reducing all proposed P450-FABP1 interaction kinetic parameters to a value of zero, as shown in Figure S16. The predictions of the direct transfer hypothesis (i.e., that FABP1 interacts with P450 3A4 to deliver substrates) was generated utilizing all experimentally determined parameters in Table S2 (Figure S16). The data fits were further refined (where possible) by computational simulation to determine the optimal parameter values required to explain the observed in vitro kinetic data.

The results of the in vitro catalytic assays and computational kinetic simulations of the data are presented in succeeding sections. Critically, this approach not only provides an unbiased assessment of whether in vitro data are supported by a proposed kinetic model (i.e., by estimating the parameter values that must be true to generate an acceptable fit), but also allows that same model to be theoretically extrapolated to conditions beyond what was tested in vitro (e.g., the high physiological concentration of FABP1 present in the cellular environment).

### Effect of FABP1 on P450 3A4 Reactions

Recombinant P450 3A4 5× reconstitution premixes (complete with POR, *b*_5_, and a phospholipid/cholate mixture) were prepared for in vitro assays as previously described,^45,46^ and calibrations against testosterone 6β-hydroxylation revealed typical P450 3A4 activity (Materials and Methods).^47^ Assays for the detection of diazepam and sulfinpyrazone metabolites were developed from existing procedures, ensuring that substrate turnover did not exceed ∼15%.^48,49^

Recombinant FABP1 was added to incubations of P450 3A4 with diazepam and sulfinpyrazone, monitoring the formation of the hydroxy (temazepam) and sulfone metabolites, respectively (Scheme 3). Experiments were performed either with a fixed concentration (5 μM) of FABP1 and a variable substrate concentration (0–10 μM), or vice versa. Incubations of P450 3A4 with diazepam were not inhibited by FABP1 in the tested concentration range, even when FABP1 was present in excess of the substrate concentration. Temazepam formation was only mildly attenuated when FABP1 exceeded the diazepam concentration (5 μM, Figure 6A) and product formation was observed to be linear even at low substrate concentrations (0.5–4 μM, Figure 6C) in the presence of a high, fixed concentration of FABP1 (5 μM). Formation of the sulfinpyrazone sulfone metabolite displayed more substantial FABP1-dependent inhibition, evidenced by roughly ∼40% attenuation of product formation by excess FABP1 (Figure 6B) and apparent rescue from inhibition at excess substrate concentrations (Figure 6D).

**Figure 6:**
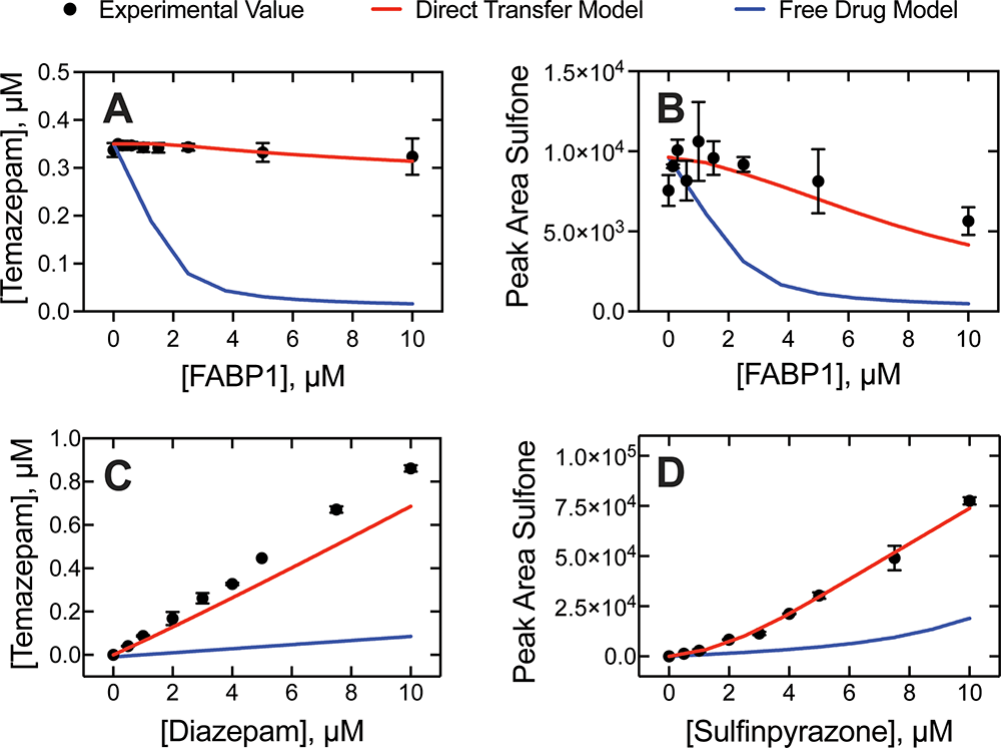
Steady-state kinetics of P450 3A4 with FABP1. FABP1 (0 to 10 μM) was added to P450 3A4 incubations (0.1 μM) containing 5 μM of (A) diazepam and (B) sulfinpyrazone. FABP1 (5 μM) was added to incubations of (C) diazepam and (D) sulfinpyrazone (0 to 10 μM). The hydroxy metabolite of diazepam (temazepam) was measured using UPLC-UV and quantified using an authentic standard curve, while the sulfone metabolite of sulfinpyrazone was detected using UPLC-MS. Plotted data (black circles) represent experimental measurements, while red and blue solid lines represent the direct transfer model and free drug model, respectively. Error bars represent the standard deviation of two replicate measurements (i.e., range).

Computational kinetic models of the experiments were rendered using experimentally determined parameters as described earlier. The data fits were further optimized by simulation-based fitting, and the adjusted parameters required to generate the modeled traces shown in Figure 6 are presented in Table S2. The effect of FABP1 on temazepam formation (from diazepam, Scheme 3) fit well to the prediction of the direct transfer model, while the free drug model predicted substantial FABP1-dependent inhibition (nearly complete inhibition by 5 μM protein) that we did not observe (Figure 6). Similarly, the free drug model predicted substantial inhibition of sulfinpyrazone oxidation by FABP1, and our data were able to be fit well to a direct transfer model.

Fitting our experimental data by simulation determined a modified suite of kinetic parameters that best fit the experimental data (Table S2). Simulation-based fitting predicted binding constants of diazepam and sulfinpyrazone to FABP1 of 0.74 μM and 0.14 μM, respectively and of 570 μM and 23 μM, respectively, to P450 3A4. The model also predicted differential capacities of the substrate-bound FABP1 to interact with P450 3A4. The *K*_d_ values predicted for the binding of P450 3A4 to singly (FS) and doubly (FSS) loaded diazepam-FABP1 complexes (Scheme 2) were roughly equivalent, being 4.2 μM and 2.4 μM, respectively, but were 0.2 μM and 0.25 μM, respectively, for sulfinpyrazone-FABP1 complexes. While our experimentally measured binding constant for P450 3A4 and Alexa-FABP1 was much lower (15 nM, Figure 5), that value was measured in the absence of substrate. The possibility that substrate binding by FABPs affects their affinity for other protein interactors cannot be excluded, and the modeled data are simply providing a set of parameters that fit our data and are ultimately not experimentally determined parameters.

### Effect of GST A1-1 on P450 3A4 reactions

The question of whether the cytosolic ligandin GST A1-1 competes with P450 3A4 for substrate binding was also considered, as it is known to bind drugs and constitutes ∼3–5% of the total cytosolic protein in human liver^24,25^ (an original intracellular concentration range of ∼100–160 μM when back-calculated from our cytosol preparation). GST A1-1 was recombinantly expressed in *E. coli* and purified to electrophoretic homogeneity (Figures S7,S8) following existing procedures^50,51^ (yield 8.9 μmol L^–1^ of bacterial culture). GST A1-1 did not bind ANS, so a ligand binding procedure based on attenuation of tryptophan fluorescence was developed based on existing procedures,^52^ but with an increased concentration of GST A1-1 (10 μM) for optimal sensitivity (data not shown). Recombinant GST A1-1 was assessed for bilirubin binding, a known substrate, to ensure the protein was functional and that the assay was effective, revealing a *K*_d_ value of 15 (±5) μM, which can be compared to a literature report of 5 μM (via fluorescence quenching)^53^ and values of 0.02 μM and 3.3 μM for a high and low affinity site (via circular dichroism and fluorescence quenching).^54^

The binding of diazepam and sulfinpyrazone to GST A1-1 was assessed in fluorescence titrations (Figure 7A,B), revealing weak binding constants of 260 (±130) μM and 97 (±14) μM, respectively. Incubations of P450 3A4 (0.1 μM) with both drugs (5 μM) revealed no significant effect of high concentrations of GST A1-1 (0 to 150 μM) on diazepam oxidation kinetics but was ∼2-fold stimulatory for sulfinpyrazone oxidation (Figure 7C,D). The 2.5-fold kinetic stimulation by GST A1-1 was unexpected, and the mechanism of this stimulation remains unclear.

**Figure 7:**
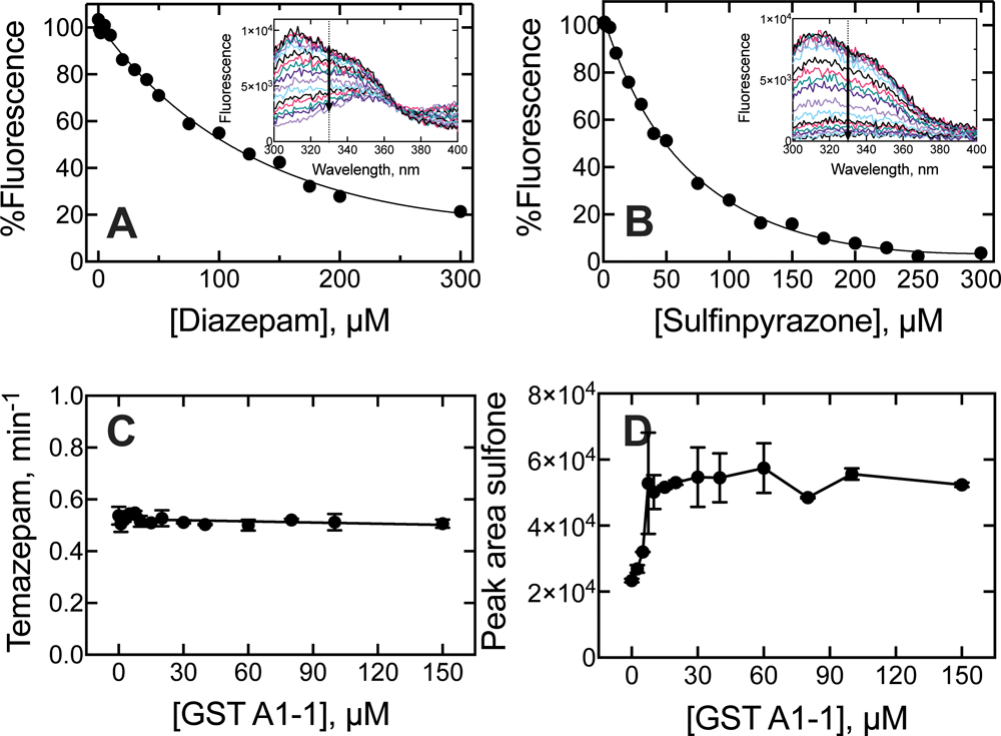
Minimal effect of GST A1-1 binding on P450 3A4 reactions. Equilibrium binding titrations of GST A1-1 (10 μM) with (A) diazepam or (B) sulfinpyrazone assessed via attenuation of tryptophan fluorescence (inset). Data points are fit to hyperbolae. Steady-state kinetics of P450 3A4 incubations with GST A1-1 (0 to 150 μM) and 5 μM (C) diazepam or (D) sulfinpyrazone. Error bars represent the standard deviation of three replicate measurements.

### Effect of Cytosol on Microsomal Drug Incubations

In order to examine the effect of FABP1 on the P450 3A4 reactions observed with recombinant protein in a more complex system, microsomal incubations were done with both drugs in the presence and absence of cytosolic protein. Microsomal and cytosolic proteins were isolated from human liver as previously described,^55^ with recovery of a ∼5.3-fold intracellular excess of cytosolic over microsomal protein. The total active microsomal P450 content was estimated at 100 pmol mg^–1^ protein via Fe^2+^-CO versus Fe^2+^ difference (Figure S9, which is low but within the typical reported range of 0.1 to 0.5 nmol mg^–1^ protein).^4^

The FABP1 content of liver cytosol was then estimated via semiquantitative polyacrylamide gel electrophoresis of cytosolic protein (Figure S10A) in comparison to a standard curve of purified, recombinant FABP1 (Figure S10B). We estimated an FABP1 content of ∼4.4 nmol mg^–1^ cytosol, corresponding to an original intracellular concentration of 365 μM FABP1. This value aligns with a recent analysis of FABP1 expression in 61 human liver samples, finding an average cytosolic FABP1 concentration of 399 μM (range of 211 μM to 760 μM) using a quantitative proteomics approach.^23^ While earlier reports had estimated FABP1 concentration via DAUDA titration,^34^ we found this approach to yield considerably higher values (∼2-fold) than the estimation via gel electrophoresis (728 μM via DAUDA titration, or ∼8.8 nmol FABP1 mg^–1^ cytosol). Our value was estimated from the apparent saturation of DAUDA binding to cytosolic protein and technically falls within the realm of some literature estimates (700–1000 μM).^14^ However, as we are unaware whether components of cytosol (other than FABP1) can bind DAUDA or otherwise confound the fluorescence assay (DAUDA displacement), we utilized the value estimated from semiquantitative SDS-PAGE (365 μM) that was also supported by the Isoherranen group’s work in all of our kinetic modeling.

The addition of cytosol to microsomal incubations of diazepam did not appear to substantially alter product formation below 2 mg mL^–1^ (∼8.8 μM cytosolic FABP1) though product formation was attenuated at higher concentrations (10 mg mL^–1^, ∼44 μM FABP1) (Figure 8). Low concentrations of cytosol (<2 mg mL^–1^, ∼8.8 μM cytosolic FABP1) stimulated sulfinpyrazone oxidation but this effect saturated at higher concentrations (Figure S15), an effect that was also observed with the addition of GST A1-1 (Figure 7). The possibility exists that cytosolic GST A1-1 may stimulate the P450 3A4 reaction through an unknown mechanism, although the enhancement with cytosol was more pronounced than with purified GST A1-1.

**Figure 8:**
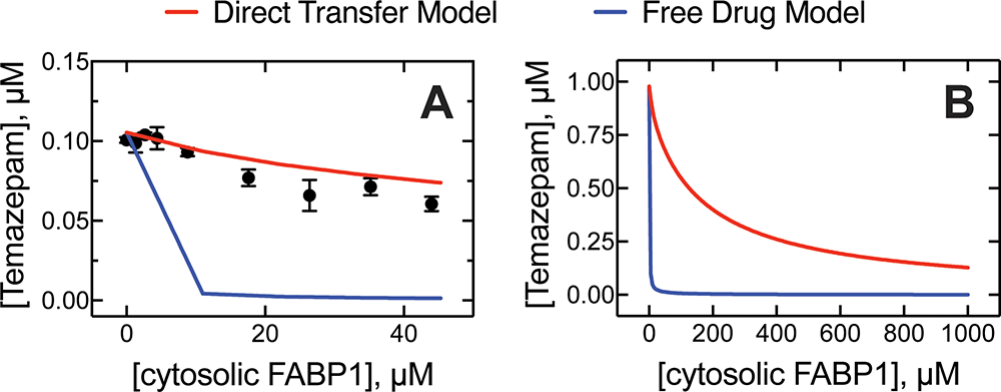
Effect of cytosol on microsomal diazepam metabolism. FABP1 constitutes roughly 7% of the total protein content of liver cytosol, representing roughly 4.4 nmol mg^–1^ (∼365 μM) in our preparation, assessed via semiquantitative SDS-PAGE analysis. (A) Cytosol (0 to 10 mg mL^–1^, 0 to ∼44 μM FABP1) was added to incubations of 5 μM diazepam with human liver microsomes (0.5 mg). Plotted data (black circles) represent experimental measurements, while red and blue solid lines represent the direct transfer model and free drug model, respectively. Error bars represent the SD of three replicate measurements. (B) The kinetic modeling in (A) was extrapolated to physiologically relevant concentrations of FABP1 (400 or 1000 μM) and P450 3A4. The reaction time was reduced to 5 min to reduce turnover to ≥20%.

We note that the preparation of microsomes and cytosol from human liver may result in a certain amount of protein from one fraction contaminating the other (i.e., cytosolic protein present in microsomes, and vice versa). We measured enzyme activities by published procedures (Materials and Methods) and estimated a 3.6% and 3.8% contamination of microsomes by cytosolic proteins (assayed for lactate dehydrogenase^56^ and glutathione transferase,^57^ respectively) and a 7.1% contamination of cytosol by microsomal protein (assayed for POR^58^). Based on these results and our (above) estimation of cytosolic FABP1 content (4.4 nmol mg^–1^ cytosol), we estimate the level of FABP1 contamination in microsomes to be roughly ∼0.16 nmol mg^–1^ microsomal protein. Given the typical concentration of microsomal protein (1 mg^–1^ mL) used in our incubations, this corresponds to a microsomal FABP1 concentration of ∼0.16 μM. As we did not observe a significant effect of submicromolar concentrations of recombinant FABP1 on P450 3A4 activity (Figure 6), nor was microsomal P450 3A4 activity substantially altered by low concentrations of cytosol (Figure 8), we find this to be a negligible level of contamination that does not interfere with the conclusions of this study.

The experimental data (Figure 8) were fit by computational modeling as described earlier, using the same kinetic parameters that were used to fit diazepam oxidation in Figure 6. The competition model predicted complete FABP1-dependent inhibition of diazepam oxidation (when the FABP1 concentration was above 20 μM) (Figure 8). The direct transfer model more closely fit our observed experimental data, using the modified rate constants presented in Table S2. The computational kinetic model of diazepam oxidation was then extrapolated to predict the effect of FABP1 on the P450 3A4 reaction at a physiologically relevant concentration of both proteins (i.e., in hepatocytes). Our experiments indicated that diazepam oxidation was attenuated mildly in the presence of recombinant FABP1 (up to 10 μM, Figure 6) and more substantially (∼40%) in the presence of cytosol (∼44 μM FABP1, Figure 8). Extrapolation of the direct transfer model also predicted attenuation, reaching nearly ∼85% inhibition of diazepam hydroxylation at a physiological concentration of 400 μM FABP1 (Figure 8). The extrapolated free drug model again predicted nearly complete inhibition of enzyme activity at an FABP1 concentration above 20 μM, as in Figure 8A.

The prediction by kinetic simulation that physiological concentrations of FABP1 will significantly slow diazepam oxidation when the substrate is delivered to P450 3A4 by FABP1 may be best explained as a result of competition among FABP1 species for P450 binding. While FABPs are known for binding fatty acids, the cytosolic medium to long chain fatty acid concentration is estimated ≤50 μM,^59^ meaning that a substantial fraction of cytosolic FABP1 (≥350 μM) is likely unliganded in the hepatocyte. In consideration of the physiological relevance of this to diazepam oxidation, we can begin by considering that a typical dose of diazepam is ∼2–10 mg (2× to 4× daily).^60^ A 10 mg dose dissolved in ∼5 L of blood would yield a total drug concentration of ∼7 μM, meaning that oral administration of a typical diazepam dose does not likely exceed the low μM range in the plasma (note: diazepam is 98% plasma protein-bound, with an albumin *K*_d_ value of ∼2 μM). Once in the hepatocyte, a very small fraction of the cytosolic FABP1 content will be required to complex the diazepam dose (with an FABP1 *K*_d_ value of 2.3 μM), meaning that FABP1-diazepam complexes are in potential competition for P450 binding with unliganded FABP1 as well as other species of FABP1 bound to diverse molecules, which may include lipids and steroids. Competition of FABP1 complexes among themselves for P450 binding may then cause an overall attenuation of P450 oxidation rate, particularly at large excesses of FABP1 over the substrate concentration, such as what we observed here with liver cytosol experiments and kinetic modeling of physiological FABP1 concentrations (Figure 8).

The prediction that the competition for substrate between FABP1 and P450 3A4 could culminate in a ∼5-fold reduction of diazepam oxidation is significant, in that this figure compares to the relative decreased clearance of some P450 3A4 substrates in hepatocytes relative to microsomes (the “CYP3A4 anomaly”).^2,7,8^ Such inhibition could be expected to impact diazepam pharmacokinetics, with the most affected parameters being overall half-life of the drug (*t*_1/2_) and intrinsic clearance. Interestingly, this effect was substrate-dependent, in that while we were able to apply a direct transfer model to sulfinpyrazone oxidation, the same kinetic parameters did not apply. Additionally, the extent of reaction inhibition with sulfinpyrazone was greater, indicating that the binding affinity of drugs to FABP1 and interaction rates of that complex with P450 are key parameters in assessing the magnitude of the FABP1-dependent effect on drug metabolism.

It is worth noting that many drugs do not bind as tightly to FABP1 as the candidates tested here (*K*_d_ < 2.5 μM). Of the 13 molecules we screened for FABP1 binding, seven demonstrated binding affinities in the range of 5 to 36 μM (Table 1). While we clearly demonstrated that the oxidation kinetics of the more tightly bound series are expected to be substantially altered at physiological concentrations of FABP1, a recent report by the Isoherranen group suggests that weaker affinity to FABP1 may still alter drug metabolism in vivo, in that their simulations revealed that FABP1 *K*_d_ values of 5 to 20 μM still compromised accuracy of predicted hepatic drug distribution.^23^ Interestingly, we did not observe strong binding of midazolam to FABP1 (*K*_d_ 21 μM), and a particularly extreme disparity has been reported between hepatocytes and liver microsomes (5-fold, but estimates have been as large as 9-fold) for the drug. Clearly, evidence suggests that FABP1-binding alone cannot fully explain the discrepancies noted as the “CYP3A4 anomaly.”

Interestingly, the binding of drugs to microsomes (whether specific or nonspecific, to microsomal membranes or protein) has been reported to affect enzyme kinetic parameters (*K*_d_, *K*_m_, or *K*_i_ but not *V*_max_).^61–63^ The presence of cytosolic protein has also been reported to alter metabolism, with notable cases being the stimulation of *N*,*N*-dimethyl-4-aminoazobenzene oxidation^13^ and the aforementioned “CYP3A4 anomaly” in which a reported majority of P450 3A4 substrates display higher intrinsic clearance in microsomal assays than with intact hepatocytes^2,7,8^ (i.e., suggesting an inhibitory effect of cytosolic protein). We report herein that the cytosolic component FABP1 can bind drugs and that it attenuates the rate of P450 3A4-catalyzed reactions. The effect of cytosolic components on drug metabolism may not be explicitly considered in pharmaceutical development but is accounted for in hepatocyte assays, as well as experiments performed in postmitochondrial liver homogenate (“S9 fraction,” i.e., 9 × 10^3^ *g* centrifugal fraction).^55,64^ Preparation of liver S9 generally involves cell lysis in four volumes of buffer over the gram weight of wet liver, yielding a 5-fold dilution of cellular components (as done in our own preparation of the subcellular fractions). Genotoxicity screens such as the Ames test routinely employ S9 but further dilute the cellular components 10- to 25-fold (diluting FABP1 to ∼3–7 μM).^65^ As we report here, the effect of FABP1 on drug metabolism (or reactions with non-natural substrates) may not be obvious at low concentrations of FABP1, i.e., concentrations substantially below physiological values.

Additionally, we considered the possible influence of cytosolic albumin on our results, but estimated a maximum intracellular albumin concentration of roughly ∼20 μM (assuming 2 g of albumin (66.5 kDa) dissolved in ∼1500 g (1.5 L) of liver tissue^66,67^ ), i.e., a concentration substantially lower than either FABP1 (≥400 μM) or GST A1-1 (≥100 μM). GST A1-1 did not have a substantial impact on P450 reactions and is far more abundant, although the *K*_d_ values for drug binding were quite weak (>70 μM). A *K*_d_ value of albumin for diazepam has been reported (∼10 μM^68^), and as estimated above the typical diazepam dose should yield plasma drug concentrations in the low μM range. We are also unaware how the presence of competing lipids (e.g., fatty acids) in cytosol may affect (or, outcompete) the weaker binding of xenobiotics such as diazepam. Given the relatively low concentration of albumin and the calculations above, we expect the effect of cytosolic albumin on our results to be minimal.

Based on our results, we position that there may be value in screening drug candidates in liver S9 fractions rather than liver microsomes for P450 3A4-metabolized substrates. As mentioned in the introductory section, the cost of hepatocytes and additional considerations regarding permeability and efflux parameters present barriers to their routine use in early screening assays. Based on our results here with FABP1 and our review of the “CYP3A4 anomaly,” we note that liver S9 retains the cytosolic protein complement of hepatocytes but is a simple postmitochondrial homogenate (lacking cell membranes and organelles), thus avoiding the cost and additional considerations of hepatocyte systems. We interpret our data to indicate that cytosolic protein, such as FABP1, may be a contributing factor to the CYP3A4 anomaly and imagine that the use of liver S9 as an initial screening system for drug candidates may offer improved IVIVE of P450 3A4 reactions. This is supported by the recent development of a physiologically based liver distribution model by the Isoherranen group, which reported markedly improved distribution prediction when intracellular drug-binding to FABP1 was included.^23^ Further, it has been demonstrated that the addition of dense extracellular protein (i.e., serum, plasma,^28^ or BSA^1^) to microsomal experiments can improve IVIVE of some reactions, with potential explanations for the effect involving molecular crowding.^29^ However, others have failed to reproduce the effect.^30^ We propose that the existing protein complement of hepatic cytosol is the most logical system to incorporate into microsomal studies (as we have done here), and reiterate our proposal for the use of liver S9.

The possibility exists that the CYP3A4 anomaly is a much more pervasive issue than previously considered, in that the effect can also manifest as disparate reaction phenotyping and can be masked altogether by the contribution of non-CYP enzymes to metabolism. An interesting example of disparate reaction phenotyping (i.e., that liver microsomes vs hepatocytes produce distinct metabolite profiles) as a consequence of the CYP3A4 anomaly was recently reported by Bapiro et al.^1^ with savolitinib–a selective kinase inhibitor, cancer therapeutic, and substrate of both P450s 1A2 and 3A4. In their example, while in vivo human excretion studies and human hepatocyte experiments had identified *N*-demethylation as the dominant pathway of metabolism, their experiments in microsomes predominantly formed a metabolite via opening of the imidazole ring. Further studies with recombinant panels of P450 enzymes revealed extensive ring opening of savolitinib catalyzed by P450 3A4, and weak *N*-demethylation activity catalyzed by P450 1A2. In this example, hepatocyte incubations correctly identified the major in vivo (desmethyl) metabolite of savolitinib formed by P450 1A2, while assays with microsomes and recombinant enzymes incorrectly assigned P450 3A4. In cases where distinct metabolites have diverse properties, this initial misassignment can be create major issue for drug metabolism scientists in IVIVE and PBPK modeling. For reference, the P450 3A4 probe drug midazolam demonstrates 5-fold higher clearance in liver microsomes relative to hepatocytes, although the dominant metabolite accurately implicates P450 3A4 as the dominant enzyme in midazolam metabolism in both assay systems: i.e., a classical manifestation of the CYP3A4 anomaly.^1,69^

It is worth nothing that non-P450 metabolism can mask the effect of the CYP3A4 anomaly altogether. When drug clearance is assessed by the disappearance of substrate alone (without consideration of the rates of metabolite formation), correspondingly low clearance in hepatocytes relative to microsomes (i.e., the CYP3A4 anomaly) can be masked by the additional contribution(s) of factors present in hepatocytes (but absent in microsomes) to metabolism (i.e., “non-CYP” mediated metabolism). The contribution of aldehyde oxidase (to an AstraZeneca drug series^2^) and uridine glycosolyl transferase (UGT) (to testosterone metabolism^1^) have reportedly masked (or compensated for) the lower P450 3A4 activity in hepatocyte systems (compared to microsomes) in the overall clearance of these molecules. In this way, we are of the opinion that the CYP3A4 anomaly may be a more pervasive issue that what has been reported.

Bapiro et al.^1^ suggested a cytochrome *b*_5_ (*b*_5_) and NADH-*b*_5_ reductase system in the P450 3A4 reaction as an explanation for the altered reaction kinetics observed in both in vitro systems, after previously invoking divergent P450 activities.^2^ They reported that recombinant P450 1A2 and NADH-*b*_5_ reductase cannot support savolitinib metabolism (i.e., to form the major human metabolite) but speculate that the system can support P450 3A4 catalysis. As microsomal proteins, the *b*_5_ and NADH-*b*_5_ reductase system is present in both microsomes and hepatocytes alongside the P450 and POR system. The necessary cofactor of NADH-*b*_5_ reductase (NADH) is present in hepatocytes (while exogenous NADPH is added to microsomal assays), although the equilibrium highly favors the oxidized form NAD^+^ (the cytoplasmic ratio is reportedly between 7 to 700-fold^70^). Importantly, POR can also reduce *b*_5_,^71^ and we have shown that *b*_5_ stimulates the reaction kinetics of NADPH-supported assays with recombinant P450 3A4 and POR roughly ∼ 3-fold, although this stimulation is not the result of electron transfer.^72^ Microsomal *b*_5_ has a high reduction potential (*E*_m,7_) (−0.14 to +0.02 V)^73,74^ and the P450 3A4 reduction potential has been measured to be −0.277 V in the presence of the substrate androstenedione.^75^ Further, we have also demonstrated that the NADH-*b*_5_ reductase system only poorly supports P450 3A4 reactions (compared to the POR system, ∼5% of the rate^76^). This is also reflected in the savolitinib data of Bapiro et al., in that the higher rate of savolitinib clearance in liver microsomes was not further stimulated by the addition of the NADH-*b*_5_ reductase-activating cofactor NADH. While NADH-supported P450 reactions in microsomes have been known for ≥50 years (e.g., with benzo­[*a*]­pyrene^77^) the reactions are reported to require *b*_5_ (i.e., cannot be explained via an NADH-*b*_5_-reductase/P450 interaction).

Given that many factors have been explored as explanations for the CYP3A4 anomaly (including direct protein–protein interactions), it is worth noting that protein–protein interactions are critical mediators of P450 reactions, and the possibility that additional protein interactors might exist is not an inherent paradigm shift. Microsomal P450 enzymes depend on interactions with microsomal redox partners (POR, *b*_5_) for catalysis, and homo- and heteromeric interactions among P450 enzymes have been reported.^78^ Some P450 reactions have been reported to be modulated by progesterone receptor membrane component 1 (PGRMC1, which shares high similarity with *b*_5_) via a direct protein–protein interaction,^79,80^ although others have proposed that the interaction is with the POR redox partner.^81^ The direct interaction of retinoid binding proteins (also cytosolic intracellular lipid binding proteins like FABPs) with retinoid-metabolizing P450 27C1 ^82^ and P450 Family 26 enzymes^83–85^ has been characterized by both the Isoherranen group and our own laboratory.

We are only aware of one study that has considered the effect of FABP1 on reactions catalyzed by P450 3A4, in which the formation of two tetrahydrocannabinol (THC) metabolites by P450 3A4 was reported to be inhibited 65–75% and 76–85%, respectively, by FABP1 (20 μM).^22^ Considering that the reported FABP1 *K*_d_ values in are in the sub-μM range for THC and the 11-OH metabolite, both molecules would be expected to be tightly complexed with FABP1 in vivo. We believe that the attenuation of P450 3A4 activity observed in that study is consistent with our proposed model here, although more kinetic parameters would have to be gathered to complete any modeling of their work. We consider that their recent report of an improved physiologically based liver distribution model that reveals the critical nature of accounting for drug-binding by FABP1 in accurately predicting drug distribution is in good agreement with the results we report here.^23^

## Conclusions

Our results clearly demonstrate that FABP1 binds and transfers at least two drugs directly to P450 3A4 for oxidation. Independent experiments with both substrates at low concentrations of FABP1 were inconsistent with a pure substrate competition model but were supported, at least in part, by a model of directed transfer (Figure 6), similar to what we previously reported with FABP1 and palmitate ω-hydroxylation by P450 4A11.^19^ Further evidence for direct transfer of diazepam to P450 3A4 was provided by cytosol-fortified microsomes (Figure 8A), supporting the role of FABP1. However, kinetic simulations suggest that at higher, physiological cytosol concentrations there may be an inhibitory effect of FABP1 (Figure 8B), possibly due to a substantial amount of substrate-devoid FABP1 competing for P450-binding in the cellular environment. We believe the effect of FABP1 may offer a potential explanation for reports of discrepancies of drug clearance assessed in hepatocytes versus with microsomes.

Curiously, recombinant FABP1 and GST A1-1 exerted opposing effects on sulfinpyrazone sulfoxidation (Figures 6, 7B) but a crude cytosolic mixture rich in both proteins yielded an unexplainable kinetic stimulation (Figure S15). Further interpretation or kinetic modeling of the effect was not possible without additional characterization of the source. Given the very wide diversity of P450 3A4 substrates,^6,86^ and some of its anomalous behavior, it is not surprising that multiple phenomena may be seen in FABP1 interactions, and our results with these two substrates warrant extension to more substrates and inhibitors of this important P450, as well as other enzymes involved in drug metabolism.

## Experimental Procedures

### Materials

In general, chemicals were obtained from Millipore-Sigma-Aldrich or Thermo Fisher unless otherwise indicated. All purchased chemicals were marked with a purity of ≥95%. The fluorophore DAUDA was synthesized and characterized as previously described,^19^ and ANS was purchased from Millipore-Sigma-Aldrich.

### Proteins

Recombinant proteins were expressed in *E. coli* and purified to electrophoretic homogeneity. An SDS-PAGE gel image of all recombinant proteins examined is presented in Figure S7.

### Fatty Acid Binding Protein 1 (FABP1)

A full-length N-terminally GST-tagged, C-terminally hexahistidine-tagged FABP1 construct was expressed in *E. coli*, purified, digested (to remove GST), and delipidated as previously described.^19^

### P450 3A4 Recombinant Enzyme System

A P450 3A4 construct bearing a modified N-terminus^87^ and C-terminal hexahistidine tag^46^ was expressed in *E. coli* and purified as described.^46,88^ The final yield was 43 nmol P450 L^–1^ bacterial culture. Rat NADPH-P450 reductase (POR) and human *b*_5_ were expressed in *E. coli* and purified as described.^71,89^ Electrophoretic purity of enzymes is presented in Figure S7. P450 3A4 was reconstituted for assays with POR and *b*_5_ using a phospholipid/cholate mixture as described previously.^45,46^ Activity of the reconstitution premixes was verified (see Steady-State Kinetic Assays) by testing testosterone 6β-hydroxylation, and a rate of 9.5 (±0.3) nmol product min^–1^ (nmol P450 3A4)^–1^ was calculated.

Enzyme concentrations were determined spectrophotometrically using an OLIS-Aminco DW2a instrument (Online Instrument Systems, Athens, GA) in the split-beam mode. P450 concentrations were measured as described^58^ by monitoring the Fe^2+^-CO versus Fe^2+^ difference spectra and using an extinction coefficient Δε_450–490_ = 91,000 M^–1^ cm^–1^ (Figure S9).^90^ Quantitation of POR was performed as described,^58^ using an extinction coefficient of ε_455_ = 23,600 M^–1^ cm^–1^.^91^ The *b*_5_ concentration was measured using an extinction coefficient Δε_424–409_ = 180,000 M^–1^ cm^–1^ for the Fe^2+^ versus Fe^3+^ difference spectra.^74,92^

### Glutathione Transferase GST A1-1

The plasmid design and protein expression of N-terminally modified full-length GST A1-1 was based on existing protocols,^50,51^ with modifications. A PET24a plasmid bearing a N-terminally modified and hexahistidine-tagged GST A1-1 cDNA (GenScript) (cloned via NdeI and NotI, Figure S8) was transformed in *E. coli* BL21 (DE3) chemically competent cells (according to the manufacturer’s protocol) and colonies were grown on Luria Broth (LB) agar plates containing kanamycin overnight (16 h, 37 °C). A single colony was used to inoculate LB media (50 mL) containing kanamycin (50 μg/mL) and the culture was grown overnight (16 h, 37 °C, 200 rpm). The overnight culture (5 mL) was used to inoculate 2 × YT media (500 mL) containing kanamycin (50 μg mL^–1^) and was grown to an OD_600_ of 0.6–0.8 (37 °C, 200 rpm). Protein expression was induced with isopropyl-1-thio-β-d-galactopyranoside (IPTG, 0.2 mM) and the cultures incubated (37 °C, 200 rpm, 3 h). Cells were harvested via centrifugation (5000*g*, 4 °C, 30 min) and stored at −80 °C until processing.

Bacterial pellets (500 mL cultures) were thawed on ice and resuspended in binding buffer (20 mM potassium phosphate buffer (pH 7.4) containing glycerol (20%, v/v), KCl (0.25 M), 2-mercaptoethanol (2 mM), phenylmethylsulfonyl fluoride (PMSF, 1 mM) and one cOmplete EDTA-free Protease Inhibitor Cocktail tablet (Roche) per 50 mL resuspension. The resuspension was sonicated for a total of 10 min (30 cycles (20s on/40s off), 45% intensity) and the lysate was centrifuged (30 min, 27,000*g*, 4 °C) to remove cell debris. The supernatant was applied to TALON resin (pre-equilibrated in binding buffer), washed with binding buffer (5 column volumes), and eluted with elution buffer (20 mM potassium phosphate buffer (pH 7.4) containing glycerol (20%, v/v), KCl (0.25 M), imidazole (0.25 M), and dithiothreitol (0.2 mM) using a linear gradient of imidazole (5 to 200 mM). The purified enzyme was concentrated (to 7 mL) and dialyzed (4 × 1 L) against storage buffer (10 mM Tris–HCl (pH 7.4) containing glycerol (10%, v/v), EDTA (1 mM), and DTT (1 mM). GST A1-1 protein concentration was estimated spectrophotometrically (A_280_) using a theoretically determined extinction coefficient (ε_280_ = 20,400 M^–1^ cm^–1^, predicted by the ExPASY ProtParam tool^93^ for the full-length protein). The final GST A1-1 protein yield was 8.9 μmol L^–1^ bacterial culture.

### Human Liver Microsomes and Cytosol

Microsomal and cytosolic protein were isolated from human liver (10 pooled donor livers, roughly ∼5 g each for 50 g total) as described in detail earlier.^55^ A total of 3.2 g of cytosolic protein and 0.60 g of microsomal protein was measured via BCA assay.^94^) P450 concentration assessed as described above^90^ (Figure S9) and P450 content was 0.10 nmol P450 mg^–1^ microsomal protein by a ferrous CO vs ferrous difference spectrum. The FABP1 content of liver cytosol was estimated via polyacrylamide gel electrophoresis at 4.4 nmol mg^–1^, and back calculation estimated an intercellular FABP1 concentration of 365 μM as described below (Figure S10, See Equilibrium Binding Titrations). Preparation of cytosol involved a 5-fold dilution of cellular components in buffer during lysis which was accounted for in the calculation.

The quality of microsome and cytosol preparations was assessed via measurement and comparison of the activities of (contaminating) microsomal enzymes in cytosol (and vice versa). The microsomal content of the cytosolic proteins glutathione transferase^57^ and lactate dehydrogenase^56^ was assessed according to published procedures, compared to the corresponding cytosolic content of each protein, and a relative figure for the cytosolic contamination of our microsome preparation was estimated. The microsomal protein content of lactate dehydrogenase and glutathione transferase was 3.6% and 3.8% that of cytosol, respectively. The content of the microsomal protein POR^58^ in cytosol was 7.1% that of the value in microsomes (although the presence of endogenous NADPH in our cytosol preparation presented a challenge in establishing a baseline for the assay).

### Equilibrium Binding Titrations

#### P450 3A4 with Drugs

Substrate binding to P450 3A4 was assessed via difference spectroscopy as described previously, using 1 μM protein dissolved in potassium phosphate buffer (100 mM, pH 7.4) in 1 cm glass cuvettes.^41,42^ All substrate solutions were prepared in C_2_H_5_OH and the organic solvent composition was ≤2% (v/v).

#### FABP1: Fluorophore (ANS, DAUDA) Displacement by Drugs

Substrate binding to FABP1 (0.50 μM) was assessed via fluorophore (DAUDA, 0.25 μM) displacement in an OLIS DM-45 spectrofluorometer as described previously.^19^ For the purpose of comparison to existing literature, some drugs were also tested in fluorophore displacement assays with 8-ANS (10 μM). The use of lower (10 μM) concentrations of ANS in the experiment was found to give optimal results (rather than the typically high concentrations (70 μM) reported in the literature,^38^ and all ANS titrations were run accordingly. All substrate solutions were prepared in C_2_H_5_OH and the organic solvent composition was ≤2% (v/v).

#### GST A1-1 with Drugs

Binding titrations with GST A1-1 were performed in a spectrofluorometer monitoring attenuation of tryptophan fluorescence,^52^ but with the modification that the protein concentration was increased to 10 μM (from 1 μM) for improved sensitivity.

#### Alexa-FABP1 with P450 Enzymes

Equilibrium binding titrations of Alexa-FABP1 with P450 3A4 were performed as described previously with P450 4A11.^19^

#### Human Liver Cytosol with DAUDA

Human liver cytosol (0.11 mg mL^–1^, assessed by the BCA assay^94^) was titrated with DAUDA (0 to 5 μM) with the same instrumentation and parameters as described for FABP1 titrations above.

### Estimation of *k*_on._Rates

All experiments were performed at 23 °C in an OLIS-RSM 1000 stopped-flow spectrophotometer/spectrofluorometer, collecting data every 1 ms with slit widths of 1.24 mm (8 nm bandpass) and gratings with 400 lines/mm and 500 nm blaze. All reagent solutions contained components at twice their desired final concentrations, accounting for a 2-fold dilution in the mixing chamber.

#### P450 3A4 with Drugs

Drug binding rates to P450 3A4 were performed as described previously,^42^ using 1 μM enzyme prepared in buffer (100 mM potassium phosphate buffer (pH 7.4) containing 1% C_2_H_5_OH (v/v) and ligand solutions prepared in the same buffer (with ethanolic substrate solutions diluted to 1% v/v).

#### FABP1 with Drugs

Ligand binding rates to FABP1 were assessed via DAUDA displacement (fluorescence attenuation) as described previously.^19^

#### Alexa Fluor-488 FABP1 to P450s

Binding of Alexa-FABP1 to P450 3A4 was performed by monitoring stimulation of Alexa-FABP1 fluorescence (>495 nm) as described previously with P450 4A11.^19^

### Steady-State Kinetic Assays

#### P450 3A4

P450 3A4 was reconstituted for assays with POR and *b*_5_ using a phospholipid/cholate 5× premixture as described previously.^45,46^ The final reagent concentrations in all P450 3A4 experiments were as follows: P450 3A4 (0.10 μM), POR (0.20 μM), *b*_5_ (0.01 μM), CHAPS (0.10 mg mL^–1^)-phospholipid mixture (0.10 mg mL^–1^), reduced glutathione (3 mM), MgCl_2_ (30 mM), and potassium HEPES (50 mM, pH 7.4). In general, P450 3A4 steady-state reactions were preincubated (5 min, 37 °C) prior to initiation via addition of an NADPH-regenerating system^55^ composed of glucose 6-phosphate (10 mM), NADP^+^ (0.5 mM), and yeast glucose 6-phosphate dehydrogenase (2 μg mL^–1^) (all final concentrations). Where indicated, substrates were aliquoted (from C_2_H_5_OH stocks) and the final organic composition was ≤1% (v/v). After the desired reaction time had passed, reactions were stopped via addition of CH_2_Cl_2_ (5 mL) and centrifuged to separate layers (10^3^ *g*, 5 min). The organic (lower) layer (4 mL) was transferred to fresh glass vials and evaporated under a stream of nitrogen gas, yielding a dried residue. The residue was reconstituted in mobile phase solvent and transferred to autosampler vials for analysis.

For reactions with recombinant FABP1, the concentration range (0 to 10 μM) was achieved via dilution of protein stock solutions (272 μM) in buffer (potassium phosphate (100 mM, pH 7.4) containing NaCl (100 mM) as described previously.^19^ For reactions with recombinant GST A1-1, the concentration range (0 to 150 μM) was achieved via dilution of protein stock solutions (3.8 mM) in GST A1-1 storage buffer (10 mM Tris–HCl (pH 7.4) containing glycerol (10%, v/v)). For both GST A1-1 and FABP1, whether the protein was added to 3A4-substrate solution or whether substrate was added to P450-protein mixtures, the addition of the final component was performed during preincubation (i.e., 5 min before reaction initiation).

### Reactions with Diazepam

Incubations of P450 3A4 with diazepam were performed based on existing procedures^49^ with some modifications. The general procedure was as described above, with the addition that reactions (0.5 mL) were allowed to proceed for 15 min (37 °C) before stopping the reaction and that the dried residue after solvent evaporation was reconstituted in 120 μL of 50% aqueous CH_3_CN.

Samples (held at 25 °C) were injected (20 μL) on a Waters BEH octadecylsilane UPLC column (2.1 × 100 mm, 1.7 μm) at a flow rate of 0.4 mL min^–1^ using a Waters Acquity UPLC system. Analytes were separated using a linear gradient of (A)­H_2_O and (B) CH_3_CN as follows (all % B, v/v): 0 min, 45%; 7.5 min, 45%; 7.6 min, 70%; 8.5 min, 70%; 8.6 min, 45%; 10 min, 45%. Products (temazepam, nordazepam) were detected using a Waters Acquity photodiode array detector set at 232 nm and quantitated via comparison to a 10-point analytical standard curve of temazepam using MassLynx software (Waters).

Representative chromatograms of diazepam incubations and UPLC-UV and UPLC-HRMS spectra of metabolites are presented in Figures S1–S6.

### Reactions with Sulfinpyrazone

Incubations of P450 3A4 with sulfinpyrazone were performed based on existing procedures^48^ with some modifications. The general procedure was as described above, with the addition that reactions (0.25 mL) were allowed to proceed for 30 min (37 °C) before stopping the reaction. The quenched reactions were supplemented with an equal volume (0.25 mL) of saturated NaCl to facilitate extraction of sulfinpyrazone and its sulfone metabolite. Experiments with a sulfinpyrazone analytical standard demonstrated inefficient recovery of the substrate without the addition of salt (75% recovery, data not shown). The sample was mixed with a vortex device again (vigorously) to ensure full extraction. The dried residue after solvent evaporation was reconstituted in 100 μL of 50% aqueous CH_3_OH.

Samples (held at 25 °C) were injected (10 μL) on a Waters BEH octadecylsilane UPLC column (2.1 × 50 mm, 1.7 μm, held at 40 °C) at a flow rate of 0.3 mL min^–1^ using a Waters Acquity UPLC system. Analytes were separated using a linear gradient of (A) 0.1% HCO_2_H in H_2_O and (B) 0.1% HCO_2_H in CH_3_OH as follows (all % B, v/v): 0 min, 25%; 0.5 min, 25%; 5.5 min, 65%; 8.5 min, 65%; 8.6 min, 25%; 10 min, 25%. Column eluate was introduced into a Waters Acquity QDa (single quadrupole) online mass spectrometer operating in the ESI positive ion mode with a cone voltage of 30 V, a sampling frequency of 10 Hz, and scanning from *m*/*z* 100 to 500. Data were processed using the MassLynx software (Waters). The reaction product (sulfone) was detected as the parent ion MH^+^ (*m*/*z* 421).

Representative chromatograms of sulfinpyrazone incubations and UPLC-UV and UPLC-HRMS spectra of metabolites are presented in Figures S1–S6.

### Reactions with Human Liver Microsomes

Reactions with microsomes and cytosol followed a similar format as described for each substrate above. Reactions (1 mL for diazepam, 0.5 mL for sulfinpyrazone) were composed of human liver microsomes (1.0 mg protein mL^–1^) in buffer (100 mM potassium phosphate (pH 7.4)) containing substrate (5 μM). Dilutions of human liver cytosol (16.6 mg protein mL^–1^ stock solution) were prepared in 100 mM potassium phosphate buffer (pH 7.4) and were added to reactions during preincubation (i.e., 5 min prior to reaction initiation). Diazepam reactions were run for 20 min, and sulfinpyrazone reactions were run for 30 min. Reactions were initiated, quenched, processed, and analyzed as described above, depending on the substrate.

## Supplementary Material





## Data Availability

KinTek Kinetic Modeling: Data were imported into KinTek Explorer software (KinTek, Snow Shoe, PA)^95^ as txt files and processed using an Apple computer (operating system 11.6.2). Example screenshots of the computational setup and modeling parameters for all modeled data are presented as Figures S16,S17. All data are contained within the article and the Supporting Information section.

## Notes

This article contains Supporting Information. Chemical analyses, details of preparation and analyses of proteins, equilibrium binding and kinetics experiments, and examples of kinetic modeling (docx) (PDF)
Spreadsheet of SMILES strings (csv) (CSV)

## Abbreviations

Alexa-FABP1: Alexa Fluor-488 conjugated FABP1
ANS: 8-anilinonapthalenesulfonic acid
*b*_5_: Cytochrome *b*_5_
DAUDA: 11-dansylaminoundecanoic acid
FABP1: liver fatty acid binding protein 1
GST A1-1: glutathione transferase A1-1
IVIVE: in vitro to in vivo extrapolation
NADPH P450 oxidoreductase: POR
P450 3A4: cytochrome P450 3A4
PBPK modeling: physiologically based pharmacokinetic modeling
UPLC: ultra performance liquid chromatography
