# Supplementary material for: Interaction of Cytochrome P450 3A4 with Fatty Acid Binding Protein 1 and Relevance to Drug Metabolism
Source: J Med Chem. 2026 Mar 4;69(6):7313–28. doi: 10.1021/acs.jmedchem.5c03754 (PMC13036779; doi:10.1021/acs.jmedchem.5c03754)
Supplement: Supplementary file 1 [file jm5c03754_si_001.pdf]

Supporting Information

Interaction of Cytochrome P450 3A4 with Fatty Acid Binding Protein 1 and Relevance to Drug Metabolism

Kevin D. McCarty, Destiny L. D. Proffett, and F. Peter Guengerich*

Department of Biochemistry, Vanderbilt University School of Medicine, Nashville, Tennessee 37232-0146, United States

Table of Contents

CHEMICAL ANALYSES

Table S1. Chemical structures, formulae, and *m/z* values of critical molecules ……..…..…p. S-2
Figure S1. UPLC UV and MS analyses of diazepam and two metabolites…………………p. S-3
Figure S2. UPLC UV and MS analyses of sulfinpyrazone and a sulfone metabolite……….p. S-4
Figure S3. LC-ESI HRMS analysis of diazepam………………………………...………..p. S-5
Figure S4. LC-ESI HRMS analysis of temazepam ……………………………...………..p. S-6
Figure S5. LC-ESI HRMS analysis of sulfinpyrazone …...……………………...………..p. S-7
Figure S6. LC-ESI HRMS analysis of sulfinpyrazone sulfone ……………….…...……...p. S-8

PROTEINS

Figure S7. Polyacrylamide gel electrophoresis (SDS-PAGE) of recombinant proteins……..p. S-9
Figure S8. Plasmid map of GST A1-1 in a PET24a vector……………………………….p. S-10
Figure S9. P450 quantification via Fe^{2+}-CO versus Fe^{2+} difference spectra……..…...……p. S-11
Figure S10. Quantitation of FABP1 in human liver cytosol by SDS-PAGE……..…...……p. S-12

EQUILIBRIUM BINDING AND KINETICS EXPERIMENTS

Figure S11. Titrations of DAUDA-FABP1 with drugs ……………………………………..p. S-13
Figure S12. Titrations of DAUDA-FABP1 with carcinogens ………………..…………p. S-15
Figure S13. Titrations of DAUDA-FABP1 with steroids …….…………………..………..p. S-16
Figure S14. Titrations of ANS-FABP1 with diazepam and sulfinpyrazone ……….………p. S-17
Figure S15. Effect of cytosolic protein on microsomal sulfinpyrazone metabolism ………p. S-18

KINETIC MODELING

Table S2. Kinetic parameters used in KinTek modeling…….……………………….....p. S-19
Figure S16. KinTek modeling of P450 3A4 reactions with FABP1 and diazepam…………p. S-20
Figure S17. KinTek modeling of P450 3A4 reactions with FABP1 and sulfinpyrazone …..p. S-21

Reference……..…………………………..p. S-23

CHEMICAL ANALYSES

Table S1. Chemical names, formulae, and structures of selected molecules.

**Name** (formula)	**SMILES**	**Structure**	***m/z* [MH]$^+$**
**1** Diazepam ($C_{16}H_{13}ClN_2O$)	O=C1CN=C(C2=C(N1C)C=CC(Cl)=C2)C3=CC=CC=C3	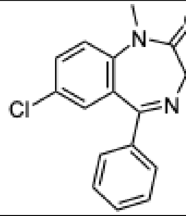	285.0789
**2** Nordazepam ($C_{15}H_{11}ClN_2O$)	O=C1CN=C(C2=C(N1)C=CC(Cl)=C2)C3=CC=CC=C3	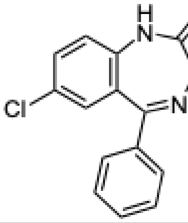	271.0633
**3** Temazepam ($C_{16}H_{13}ClN_2O_2$)	O=C1N(C2=C(C(C3=CC=CC=C3)=NC1O)C=C(Cl)C=C2)C	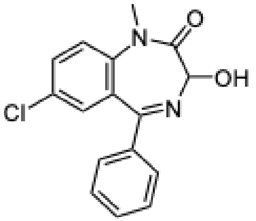	301.0738
**4** Sulfinpyrazone ($C_{23}H_{20}N_2O_3S$)	O=C(C(C1=O)CCS(C2=CC=CC=C2)=O)N(N1C3=CC=CC=C3)C4=CC=CC=C4	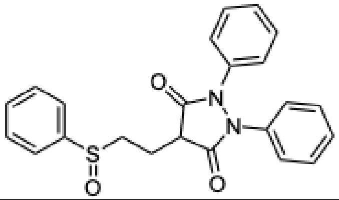	405.1267
**5** Sulfinpyrazone sulfone ($C_{23}H_{20}N_2O_4S$)	O=C(C(C1=O)CCS(C2=CC=CC=C2)(=O)=O)N(N1C3=CC=CC=C3)C4=CC=CC=C4	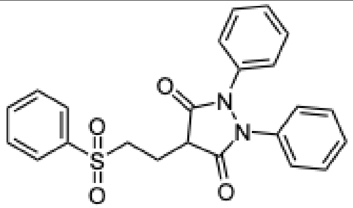	421.1217
**6** DAUDA 11-dansylamino-undecanoic acid ($C_{23}H_{34}N_2O_4S$)	O=C(O)CCCCCCCCCCNS(C1=CC=CC2=C(C=CC=C21)N(C)C)(=O)=O	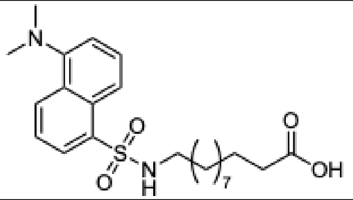	435.2312
**7** ANS (8-Anilino-1-naphthalene-sulfonic acid) ($C_{16}H_{13}NO_3S$)	O=S(C1=C2C(NC3=CC=CC=C3)=CC=CC2=CC=C1)(O)=O	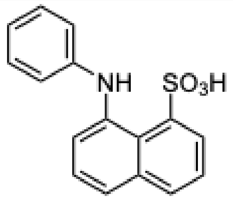	300.0689

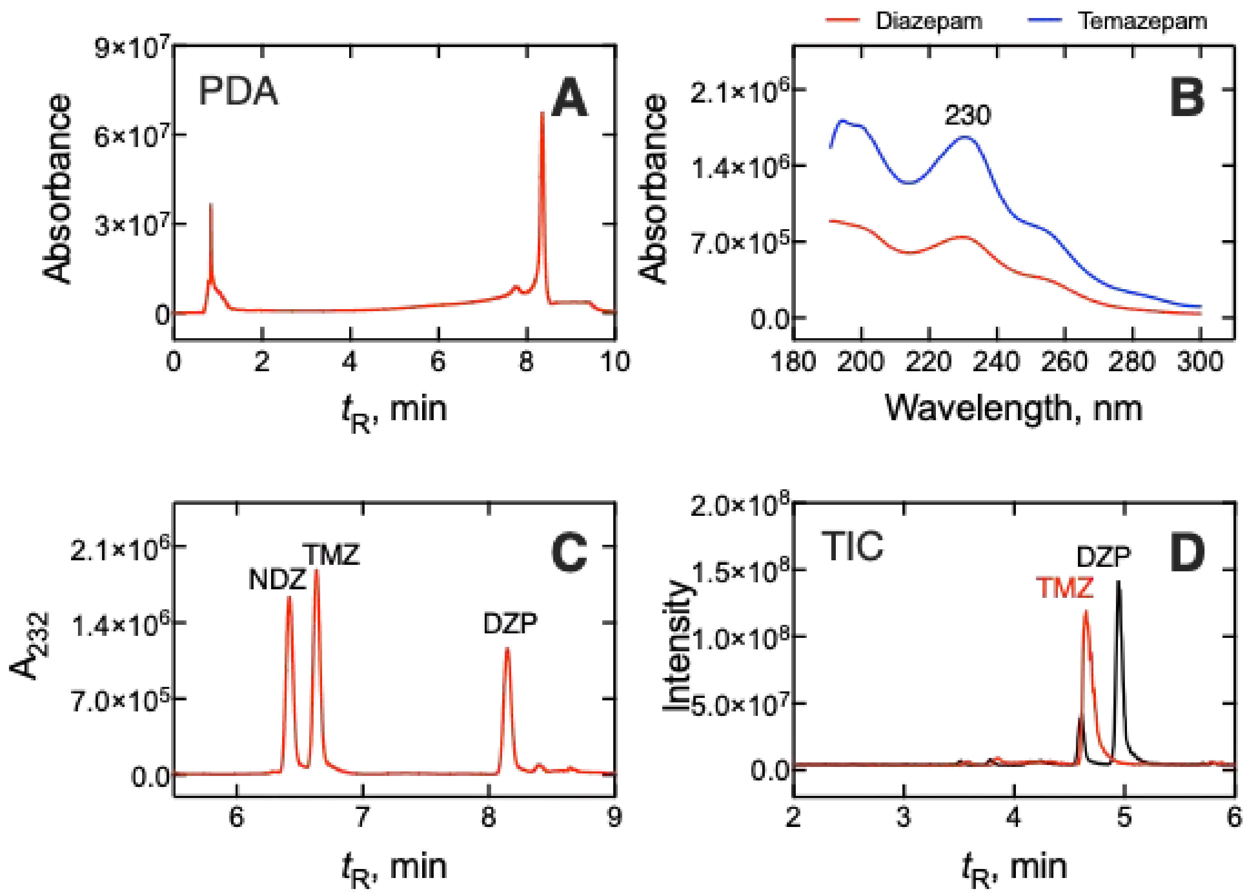

Figure S1. UPLC UV and MS analyses of diazepam and reaction products. (A) Photodiode array (192-300 nm) UPLC trace of a diazepam analytical standard (1000 pmol injected). (B) UV spectra (192-300 nm) of diazepam (red) and its P450 reaction product temazepam (blue). Spectra were obtained from the chromatogram presented in (C). 100 pmol of each standard was injected. (C) UPLC-UV chromatogram of diazepam (DZP), temazepam (TMZ), and nordazepam (NDZ) showing the separation of DZP from its two main P450 3A4 products (100 pmol of each standard was injected). (D) LC-ESI HRMS total ion current chromatogram of DZP and TMZ (1000 pmol injections). The HRMS spectra of both molecules are presented in Figures S3-S4. Absorbance is in relative units in Parts A, B, and C.

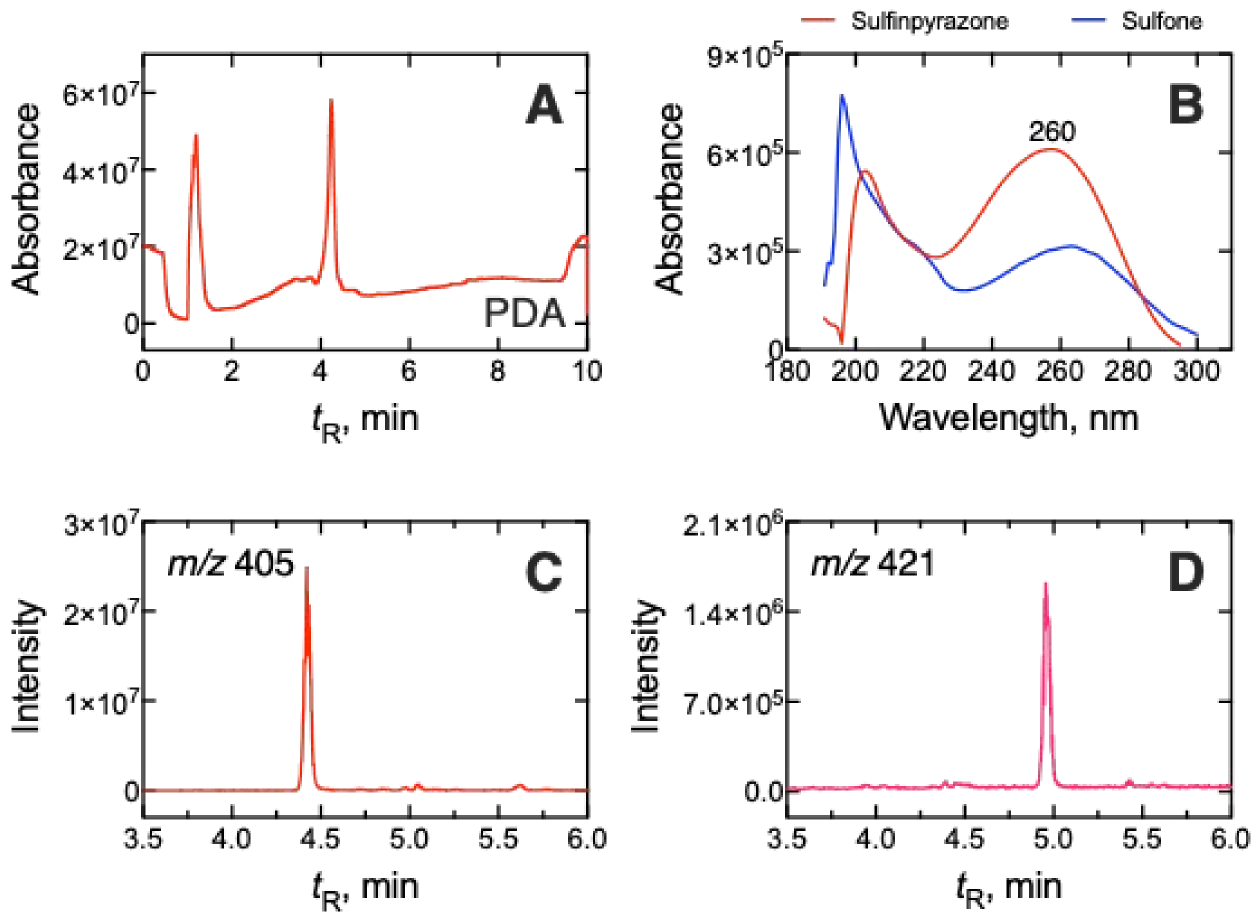

Figure S2. UPLC UV and MS analyses of sulfinpyrazone and a sulfone product. (A) Photodiode array (192-300 nm) UPLC trace of a sulfinpyrazone analytical standard (1000 pmol injected). (B) UV spectra (192-300 nm) of sulfinpyrazone (red) and its P450 reaction product, a sulfone (blue). 1000 pmol sulfinpyrazone was injected (sulfone concentration not specified). UPLC-ESI MS extracted ion chromatograms of (C) sulfinpyrazone (*m/z* 405) and (D) the sulfone oxidation product (*m/z* 421). HRMS spectra of both molecules are presented in Figures S5-S6. Absorbance is in relative units in Parts A and B

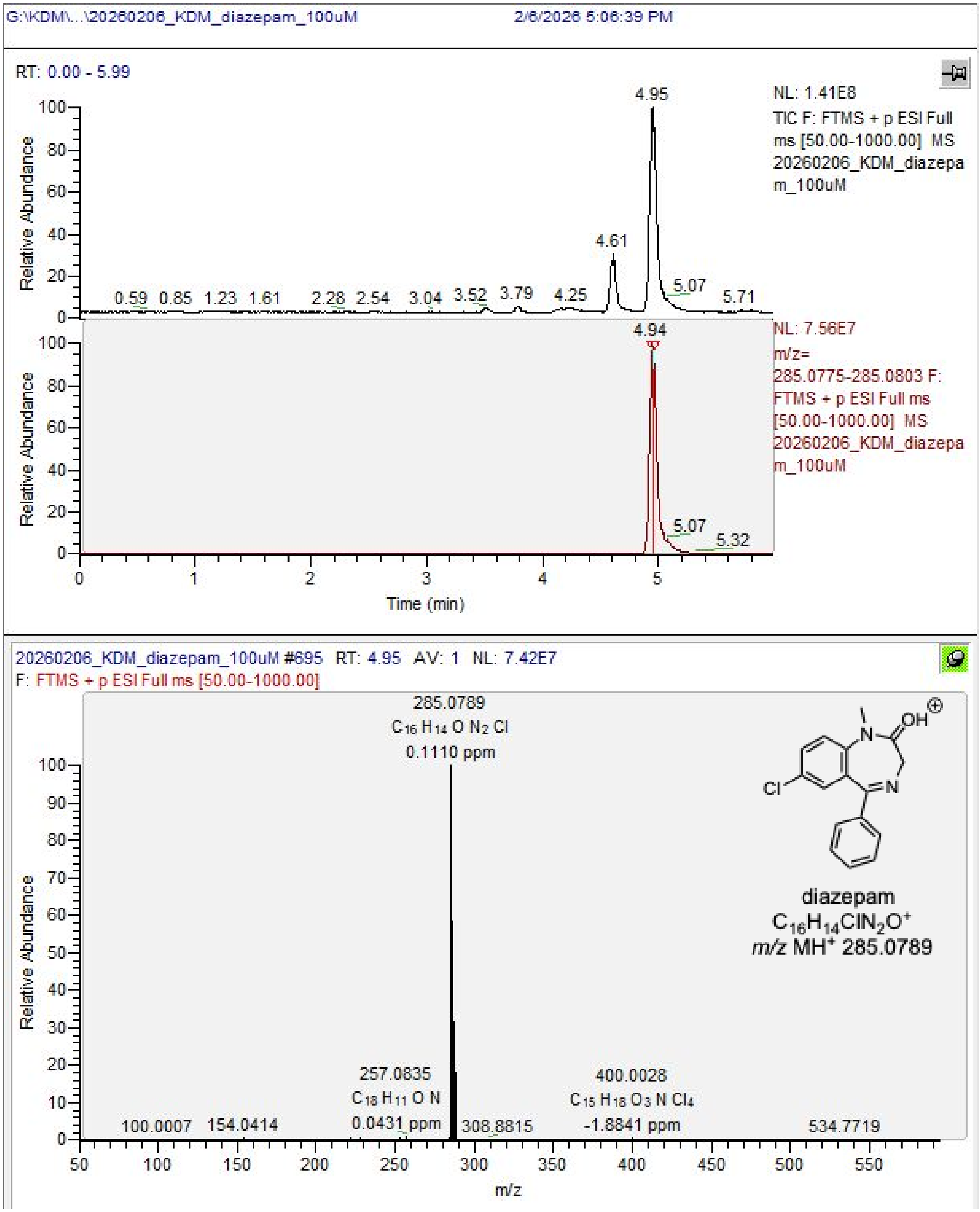

Figure S3. LC-ESI HRMS analysis of diazepam. The upper chromatogram is the total ion current and the lower is the extracted ion chromatogram displayed with a ±5 ppm *m/z* window from the diazepam MH^+ theoretical *m/z* (*m/z* 285.0789). The MS spectrum (below) is provided of the extracted diazepam MH^+ peak. 1000 pmol of the analytical standard was injected. The *m/z* Δ from theoretical was 0.1 ppm.

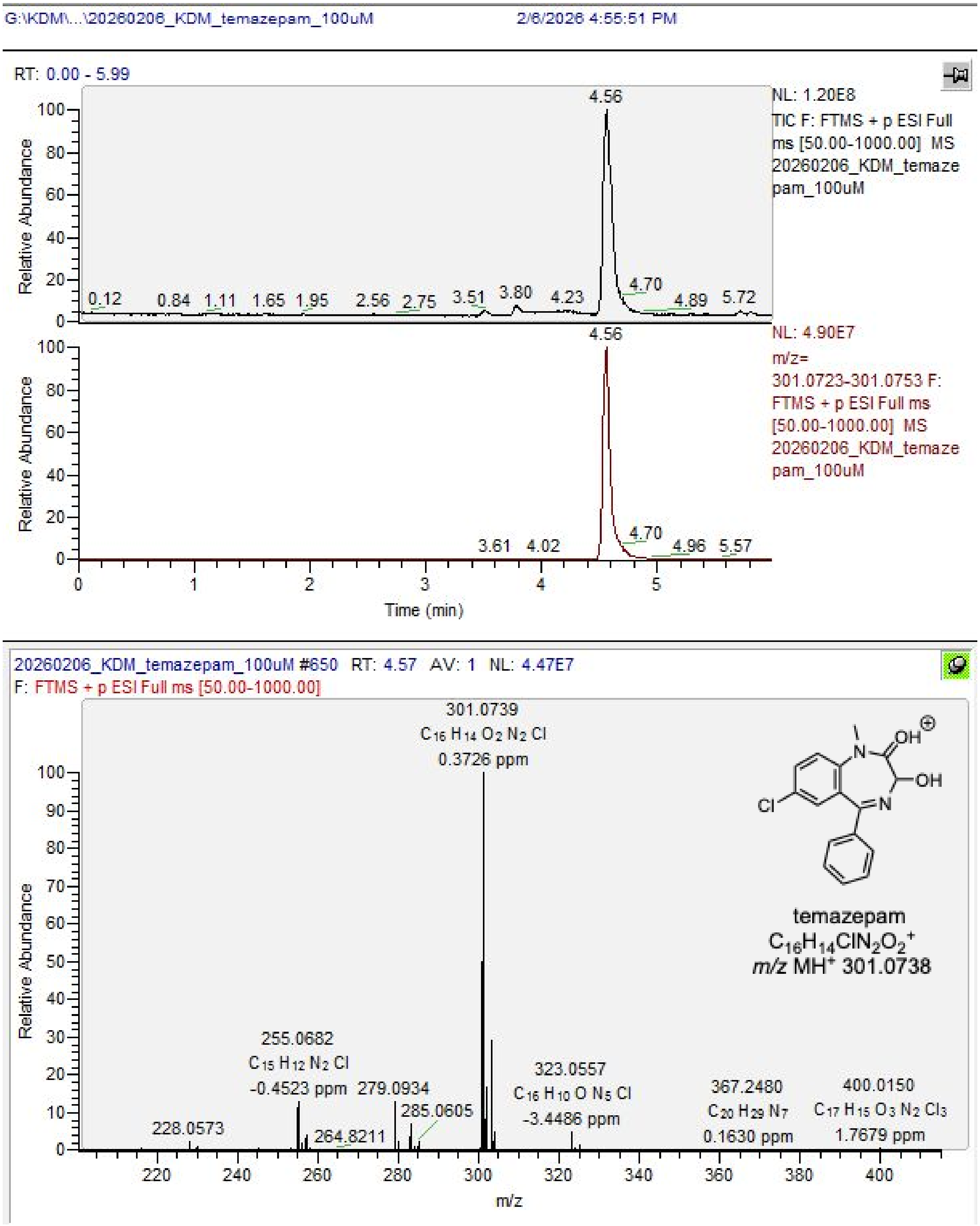

Figure S4. LC-ESI HRMS analysis of temazepam. The upper chromatogram is the total ion current and an the lower is the extracted ion chromatogram displayed with a ±5 ppm *m/z* window from the temazepam MH^+ theoretical *m/z* (*m/z* 301.0738). The MS spectrum (below) is provided of the extracted temazepam MH^+ peak. 100 pmol of the analytical standard was injected.. The *m/z* Δ from theoretical was 3.4 ppm.

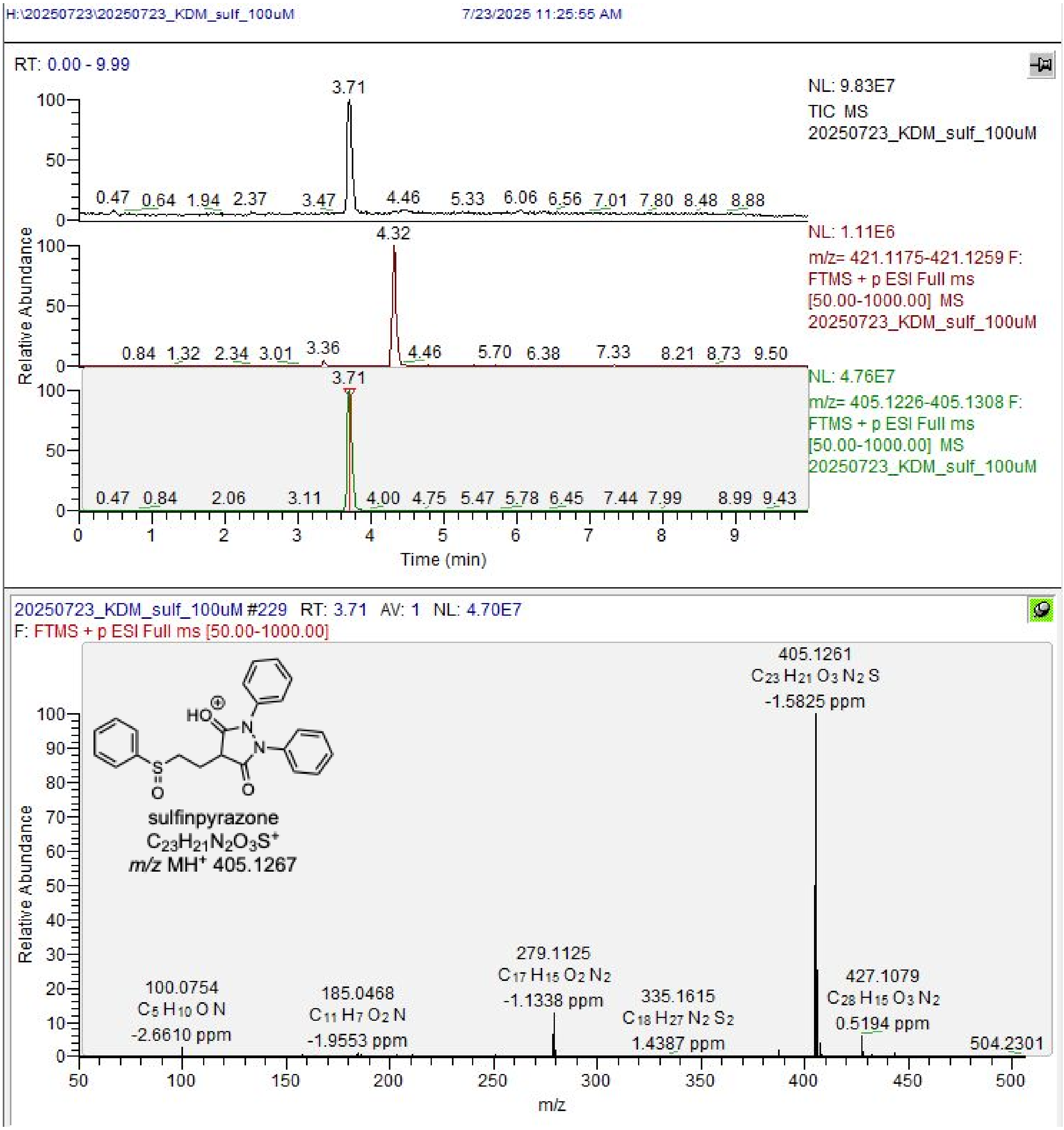

Figure S5. LC-ESI HRMS analysis of sulfinpyrazone. The upper chromatogram is the total ion current and an the lower is the extracted ion chromatogram displayed with a ±5 ppm *m/z* window from the sulfinpyrazone MH^+ theoretical *m/z* (*m/z* 405.1267). The MS spectrum (below) is provided of the extracted sulfinpyrazone MH^+ peak. 1000 pmol of the analytical standard was injected.. The *m/z* Δ from theoretical was 1.6 ppm.

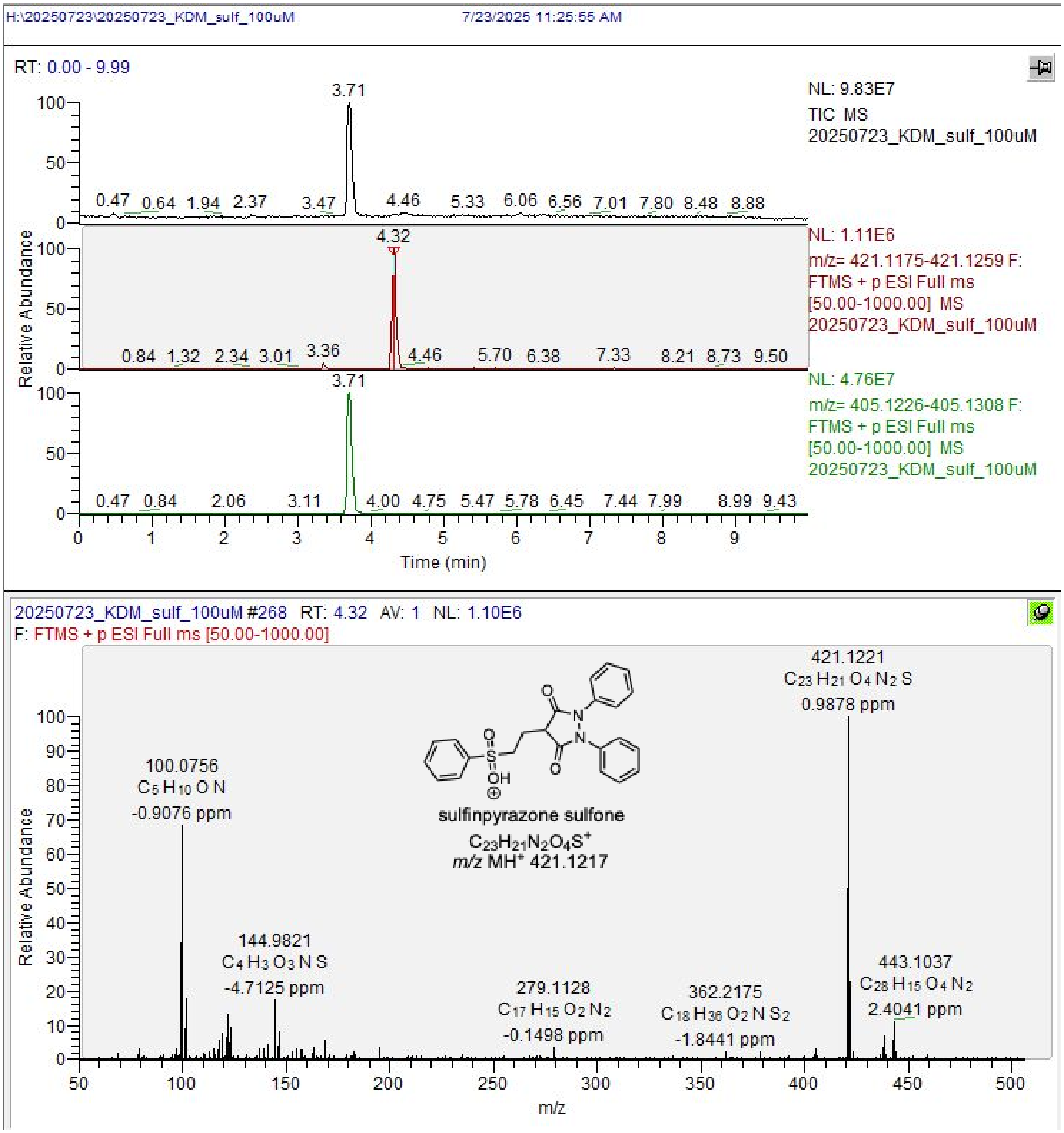

Figure S6. LC-ESI HRMS analysis of sulfinpyrazone sulfone product. The upper chromatogram is the total ion current and an the lower is the extracted ion chromatogram displayed with a ±5 ppm *m/z* window from the sulfone MH^+ theoretical *m/z* (*m/z* 421.1217). The MS spectrum (below) is provided of the extracted sulfinpyrazone MH^+ peak. The concentration of the product sulfone was not specified. The *m/z* Δ from theoretical was 1.0 ppm.

PROTEINS

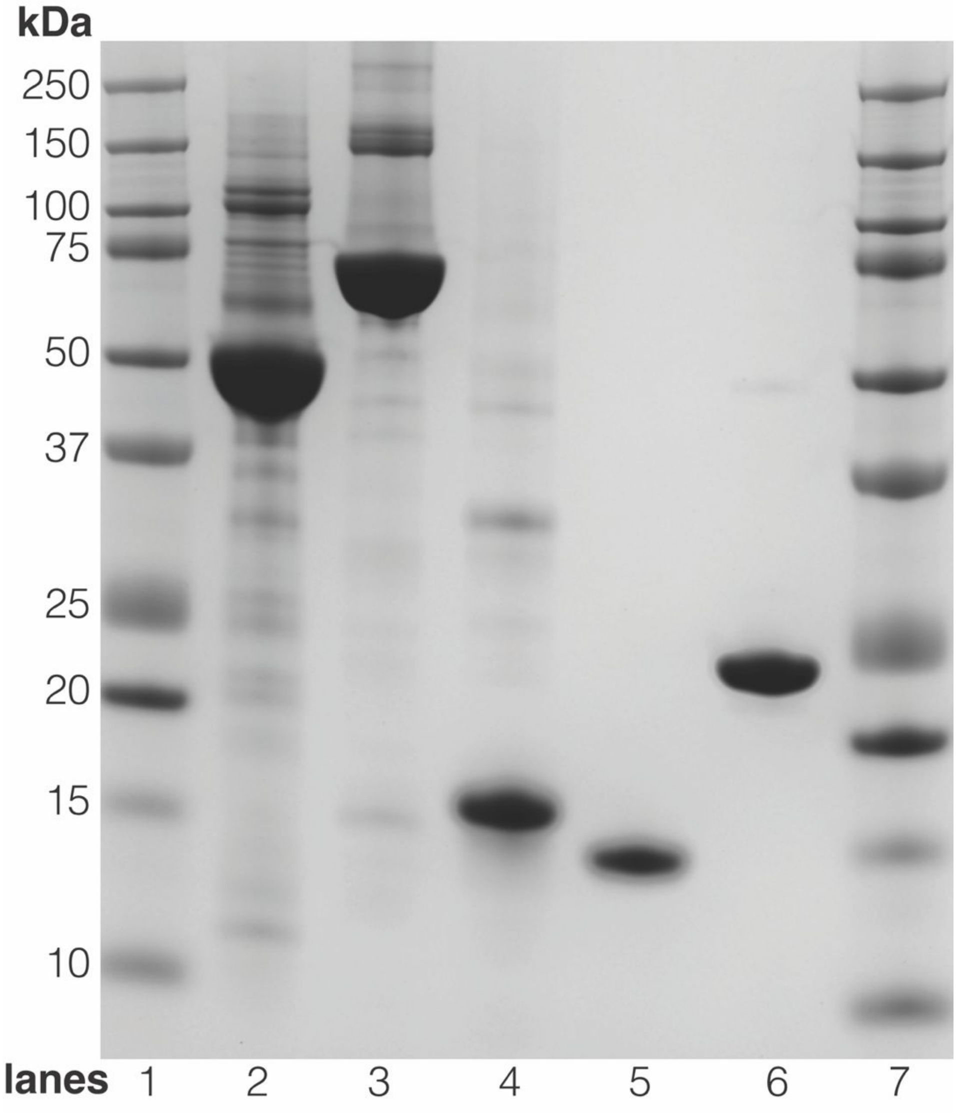

Figure S7. Polyacrylamide gel electrophoresis (SDS-PAGE) of purified recombinant proteins. A Precision Plus Protein Kaleidascope ladder was loaded in the first (lane 1) and last (lane 7) lanes. P450 3A4 (lane 2), NADPH-P450 reductase (lane 3), cytochrome b_5 (lane 4), FABP1 (lane 5), and GST A1-1 (lane 6) were denatured in Laemmli loading dye[1] heated at 98 °C (10 min), and loaded (100 pmol) on a NuPAGE 4-12% Bis-Tris polyacrylamide gel (w/v) developed in K^+MOPS for 90 min at 130 V. Proteins were stained with SimplyBlue SafeStain and destained in MilliQ H_2O.

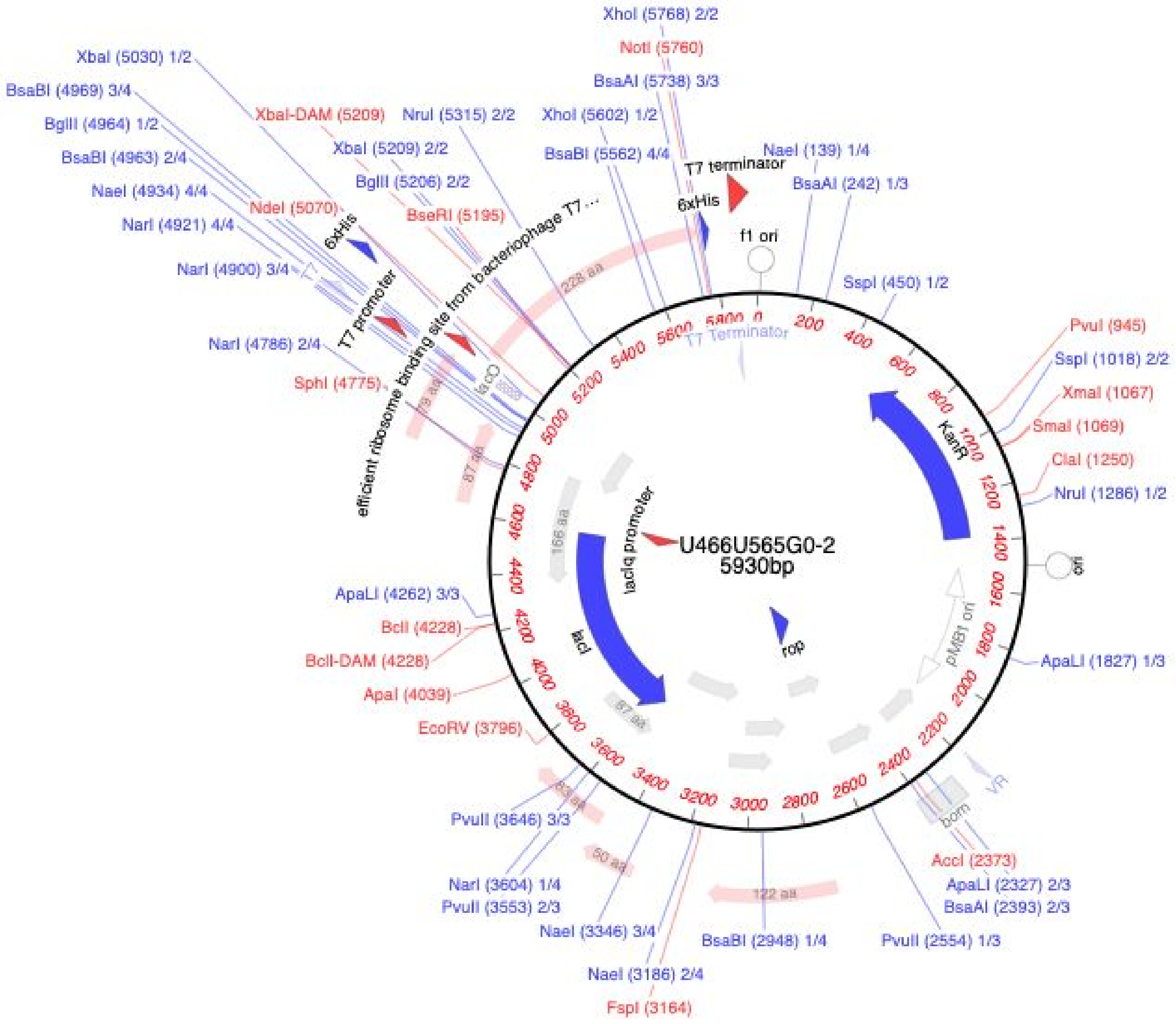

Figure S8. Plasmid map of GST A1-1 in a PET24a vector. The GST A1-1 gene with a modified N-terminus and N-terminal hexahistidine tag was cloned into a PET24a vector via NdeI and NotI (GenScript).

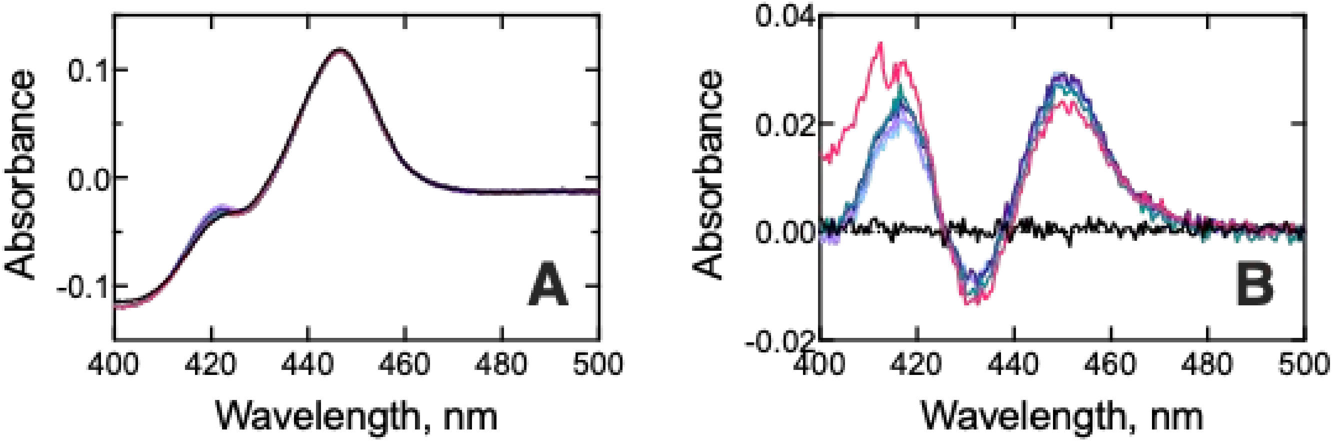

Figure S9. P450 quantification by reduced CO vs. reduced CO difference spectra. Five spectra were recorded in succession of (A) purified recombinant P450 3A4 and (B) human liver microsomes. The P450 concentration in the cuvettes was (A) 1.5 µM and (B) 0.30 µM, respectively. For Part B, the sample had been diluted such that the protein concentration (as measured by BCA assay) was 3.3 mg mL^{-1}, giving a P450 content of 0.10 nmol mg^{-1} microsomal protein.

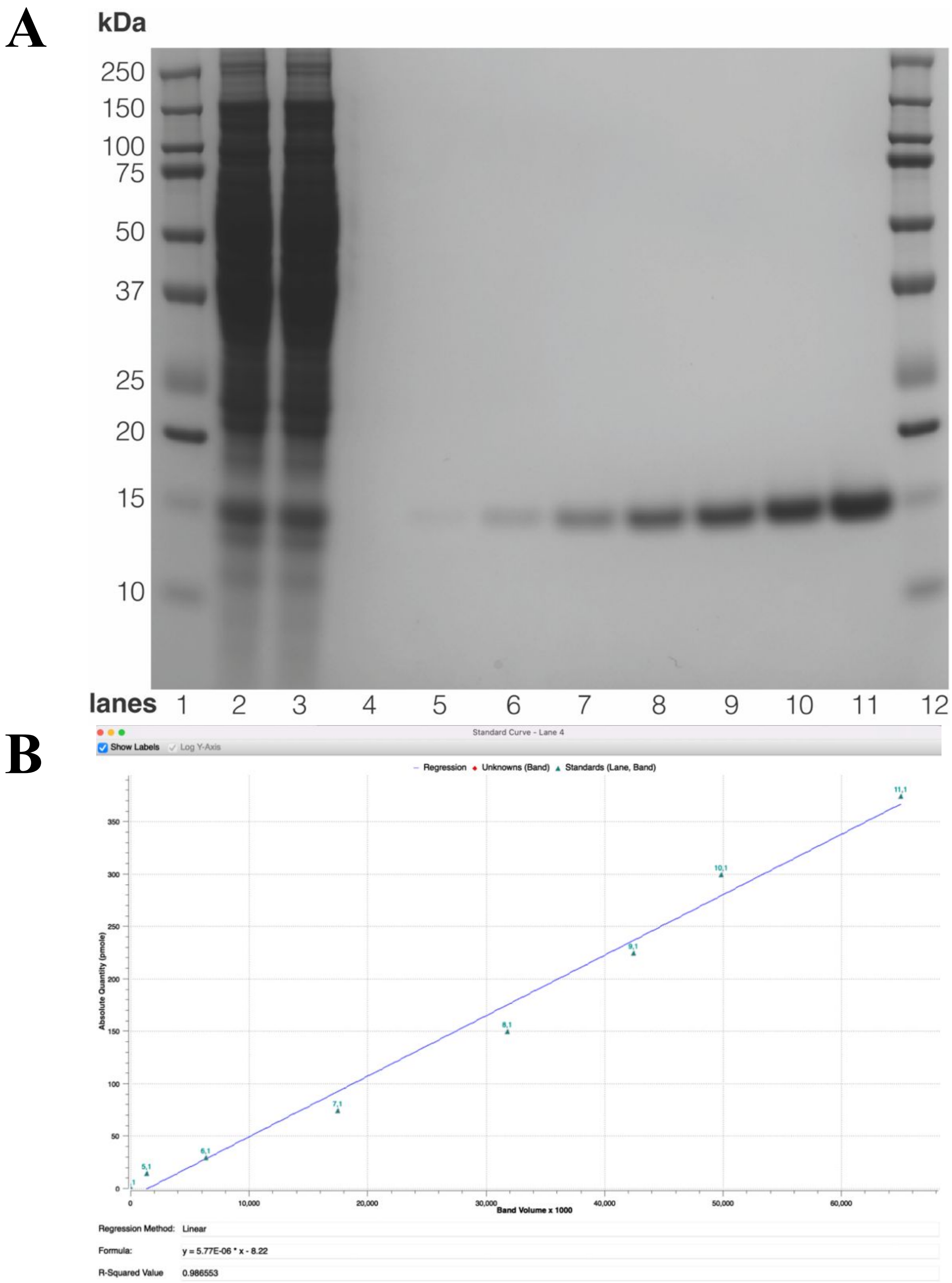

Figure S10. Quantitation of FABP1 in human liver cytosol by SDS-PAGE. (A) SDS-PAGE analysis of cytosol. A Precision Plus Protein Kaleidascope ladder was loaded in the first (lane 1) and last (lane 12) lanes. Human liver cytosol (20.8 µg, lanes 2-3) and purified recombinant FABP1 (0-375 pmol, lanes 4-11) were denatured in Laemmli loading dye,[1] heated at 96 °C (5 min), and loaded on a NuPAGE 4-12% Bis-Tris polyacrylamide gel (w/v) developed in MES buffer for 90 min at 130 V. Proteins were stained with SimplyBlue SafeStain and destained in MilliQ H_2O. (B) Standard curve of FABP1 band volume as a function of pmol recombinant FABP1 loaded o the gel (lanes 4-11, 0-375 pmol). A value of ~91 pmol (n=2) of FABP1 was calculated in 20.8 µg cytosol (4.4 nmol FABP1 mg^{-1} cytosol).

EQUILIBRIUM BINDING AND KINETICS EXPERIMENTS

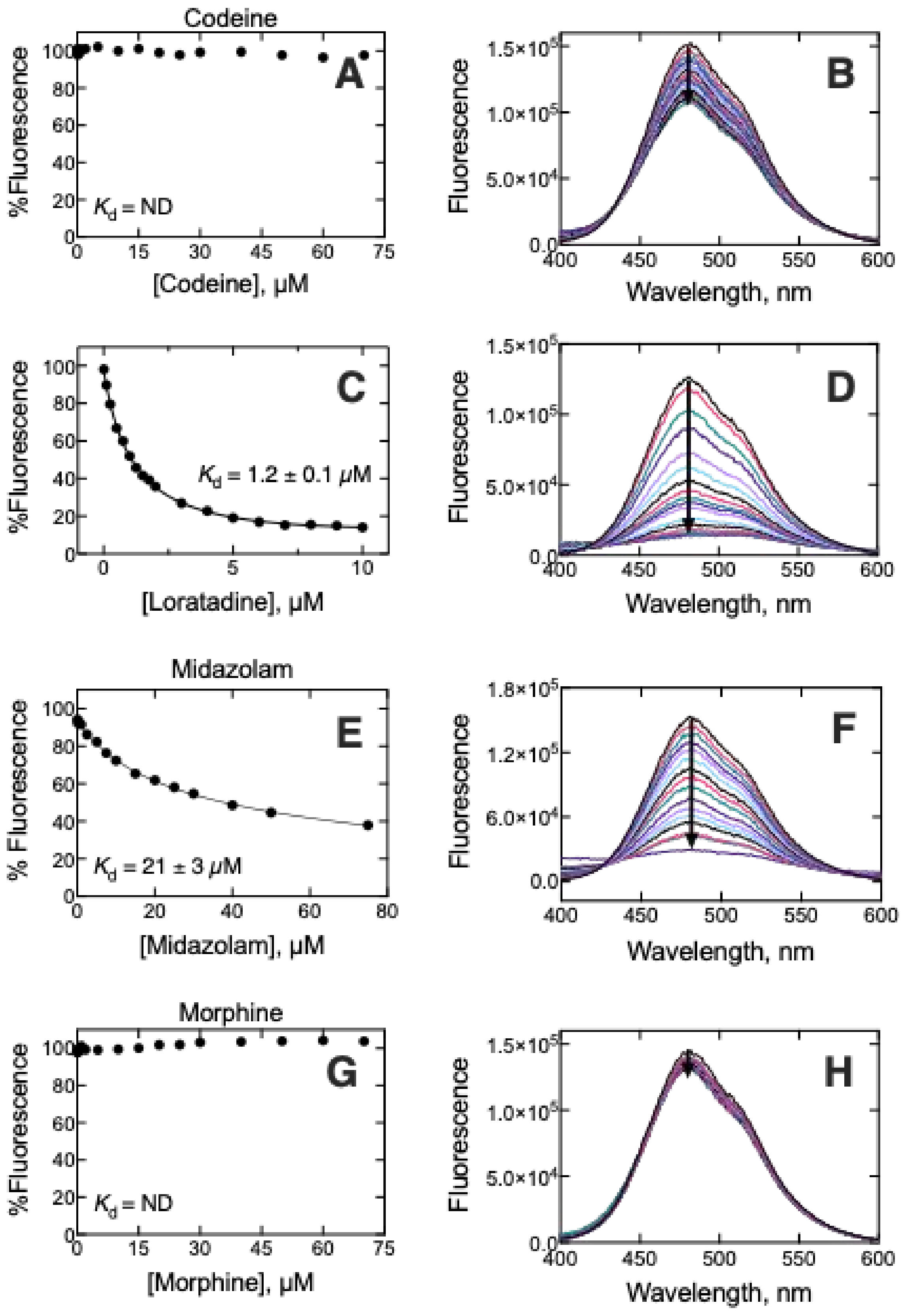

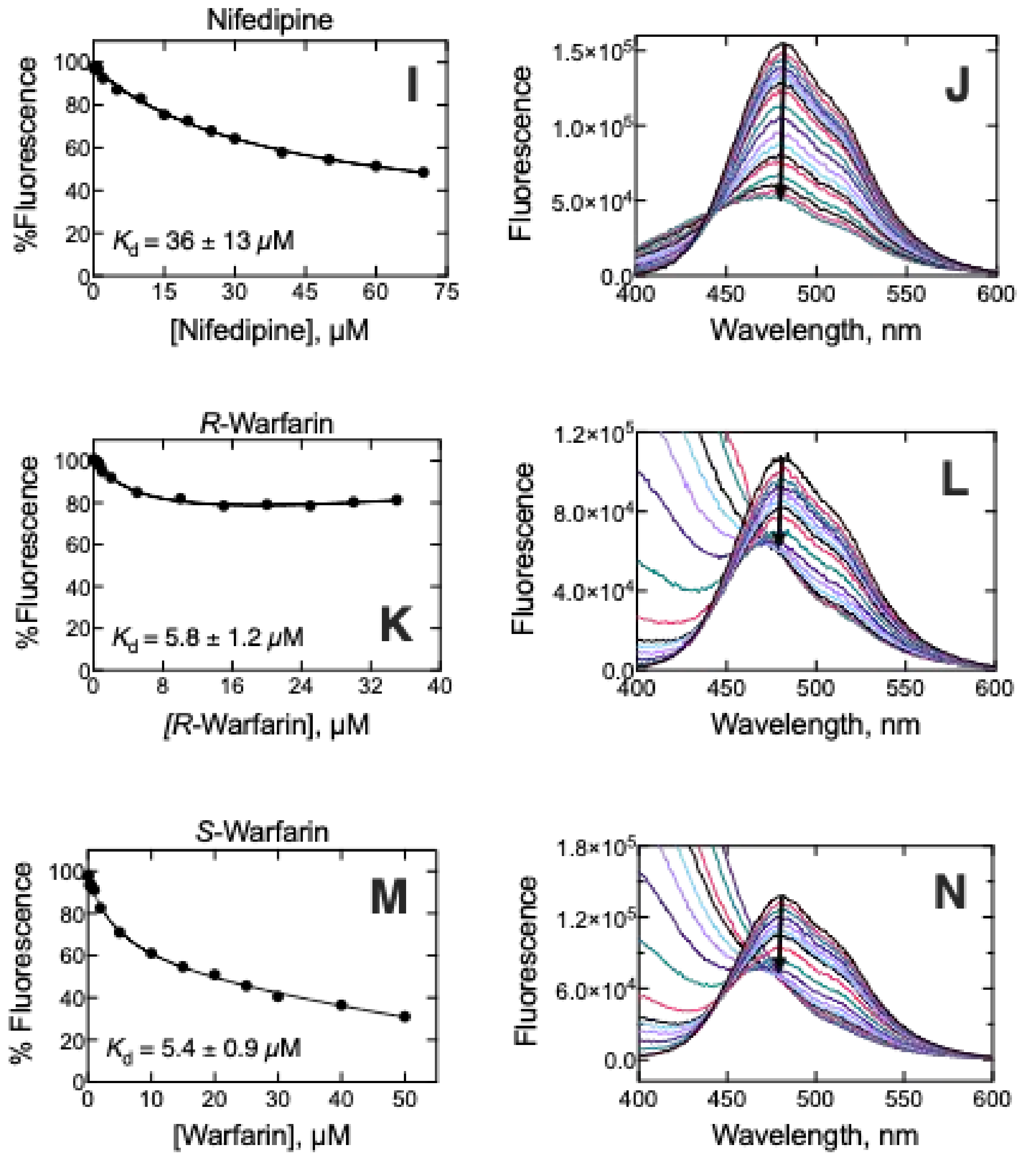

Figure S11. Equilibrium binding titrations of drug P450 3A4 substrates to DAUDA-FABP1. Fluorescence displacement curves of DAUDA-FABP1 with (A) codeine, (C) loratadine, (E) midazolam, (G) morphine, (I) nifedipine, (K) *R*-warfarin, and (M) *S*-warfarin. Corresponding fluorescence emission spectra recorded after each addition of (B) codeine, (D) loratadine, (F) midazolam, (H) morphine, (J) nifedipine, (L) *R*-warfarin, and (M) *S*-warfarin. The arrows mark the decreasing signal direction that accompanies DAUDA displacement from FABP1 and the corresponding binding of substrate.

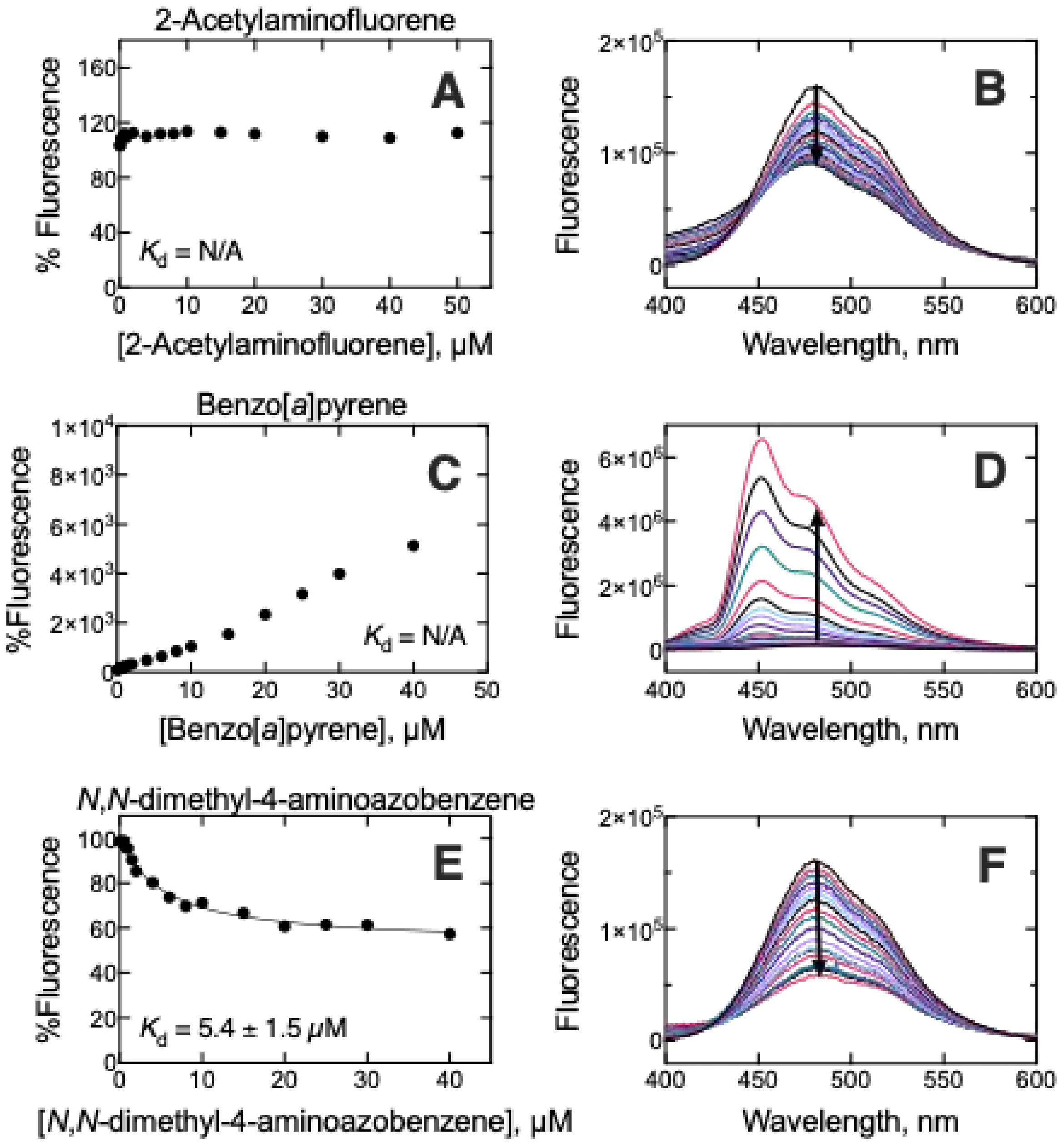

Figure S12. Equilibrium binding titrations of some P450 3A4 carcinogenic substrates to DAUDA-FABP1. Fluorescence displacement curves of DAUDA-FABP1 with (A) 2-acetylaminofluorene, (C) benzo[*a*]pyrene, and (E) *N*,*N*-dimethyl-4-aminoazobenzene. Corresponding fluorescence emission spectra recorded after each addition of (B) 2-acetylaminofluorene, (D) benzo[*a*]pyrene, and (F) *N*,*N*-dimethyl-4-aminoazobenzene. Arrows marks the decreasing signal direction that accompanies DAUDA displacement from FABP1 and the corresponding binding of substrate. Benzo[*a*]pyrene had endogenous fluorescence, resulting in an increase in emission signal intensity over the experiment.

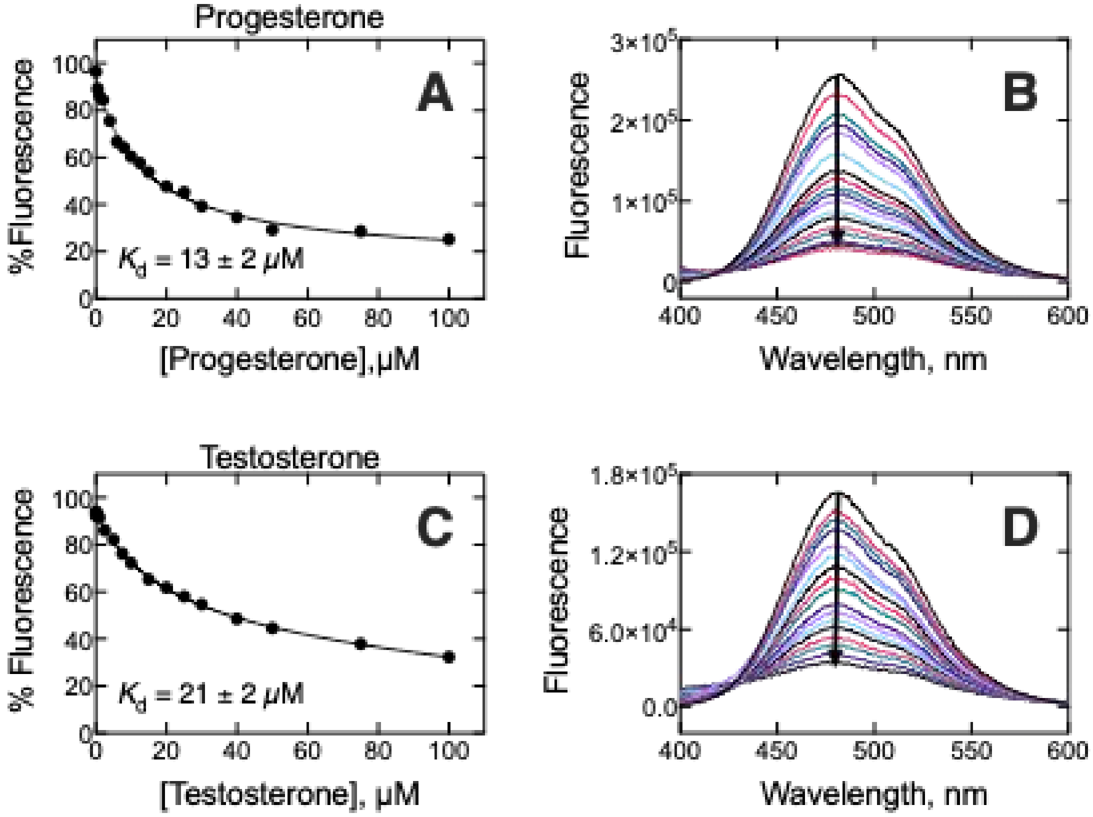

Figure S13. Equilibrium binding titrations of P450 3A4 steroid substrates to DAUDA-FABP1. Fluorescence displacement curves of DAUDA-FABP1 with (*A*) progesterone and (C) testosterone. Corresponding fluorescence emission spectra recorded after each addition of (B) progesterone and (D) testosterone. Arrows mark the decreasing signal direction that accompanies DAUDA displacement from FABP1 and the corresponding binding of substrate.

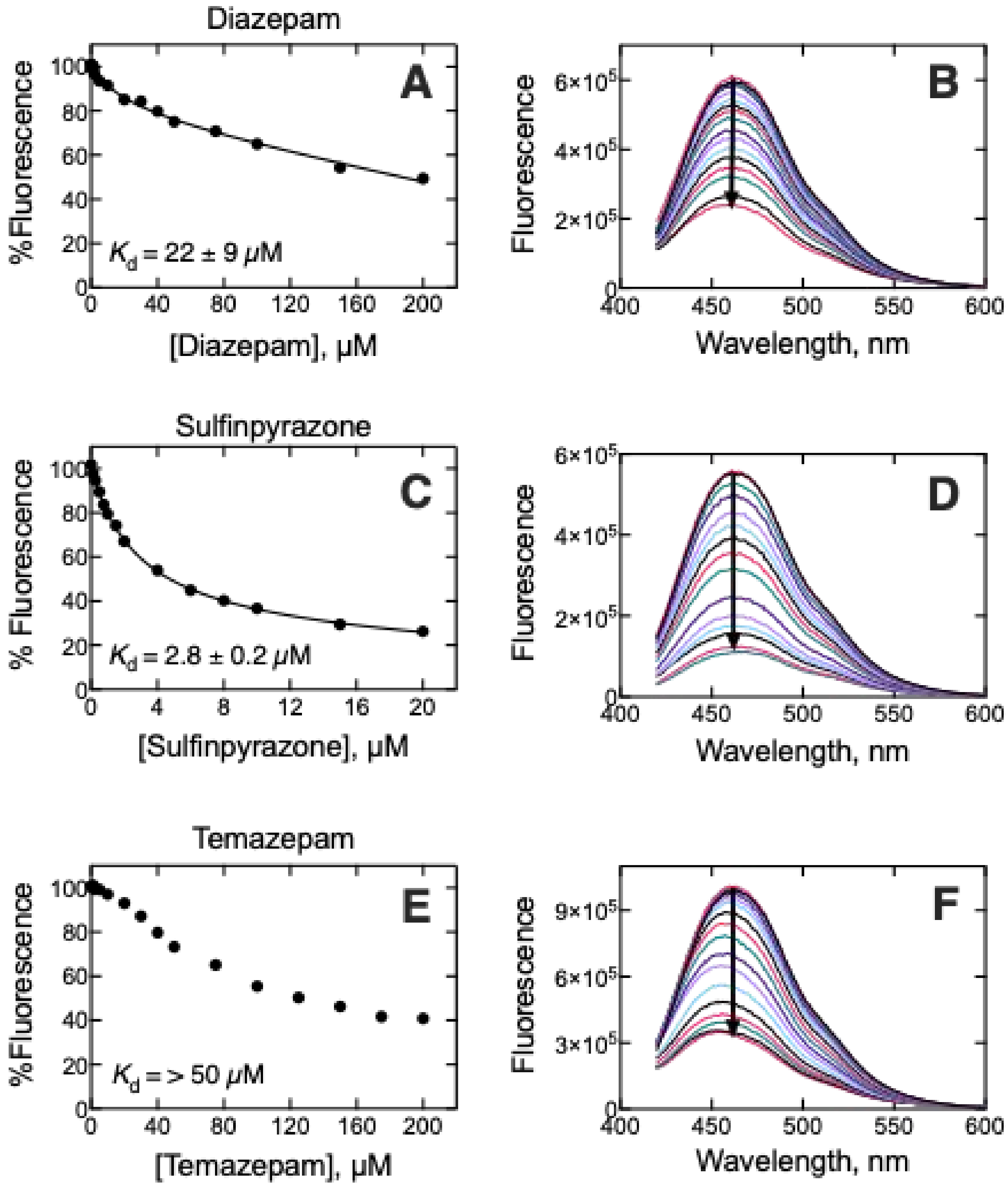

Figure S14. Equilibrium binding titrations of ANS-FABP1 with selected P450 3A4 ligands. Fluorescence displacement curves of DAUDA-FABP1 with (A) diazepam, (C) sulfinpyrazone, and (E) temazepam. Corresponding fluorescence emission spectra recorded after each addition of (B) diazepam, (D) sulfinpyrazone, and (F) temazepam. The arrows mark the decreasing signal direction that accompanies DAUDA displacement from FABP1 and the corresponding binding of substrate.

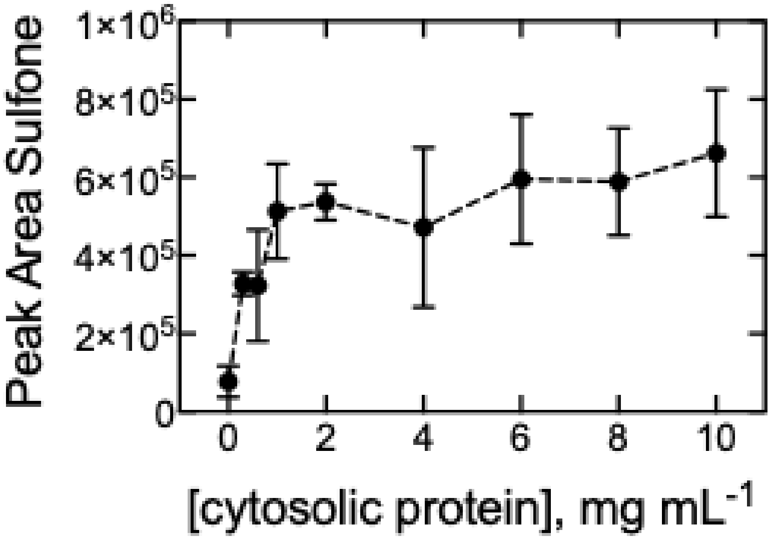

Figure S15. Effect of cytosolic protein on microsomal sulfinpyrazone metabolism. (A) Cytosol (0 to 10 mg mL^{-1}) was added to incubations of 5 μM sulfinpyrazone with human liver microsomes (0.5 mg). Plotted data (black circles) represent experimental measurements, while red and blue solid lines represent the direct transfer model and free drug model, respectively. Error bars represent the standard deviation of three replicate measurements.

KINETIC MODELING

Table S2. Kinetic parameters predicted by KinTek kinetic modeling.

Reaction	Diazepam		Sulfinpyrazone	
	k_{on}, μM^{-1} s^{-1}	k_{off}, s^{-1}	k_{on}, μM^{-1} s^{-1}	k_{off}, s^{-1}
E + S = ES	0.10	57.6	3.53	81.3
F + S = FS	0.122	0.0905	4.13	0.578
FS + S = FSS	0.294	0.0852	0.724	0.176
ES = EP	0.473	0	0.437	0
EP = E + P	25.7	0.0428	7.43	0.177
F + P = FP	0.108	3.78	3.78	20.4
E + FS = ES + F	0.084	0.356	0.483	0.108
E + FSS = ES + FS	0.203	0.495	7.19	1.7

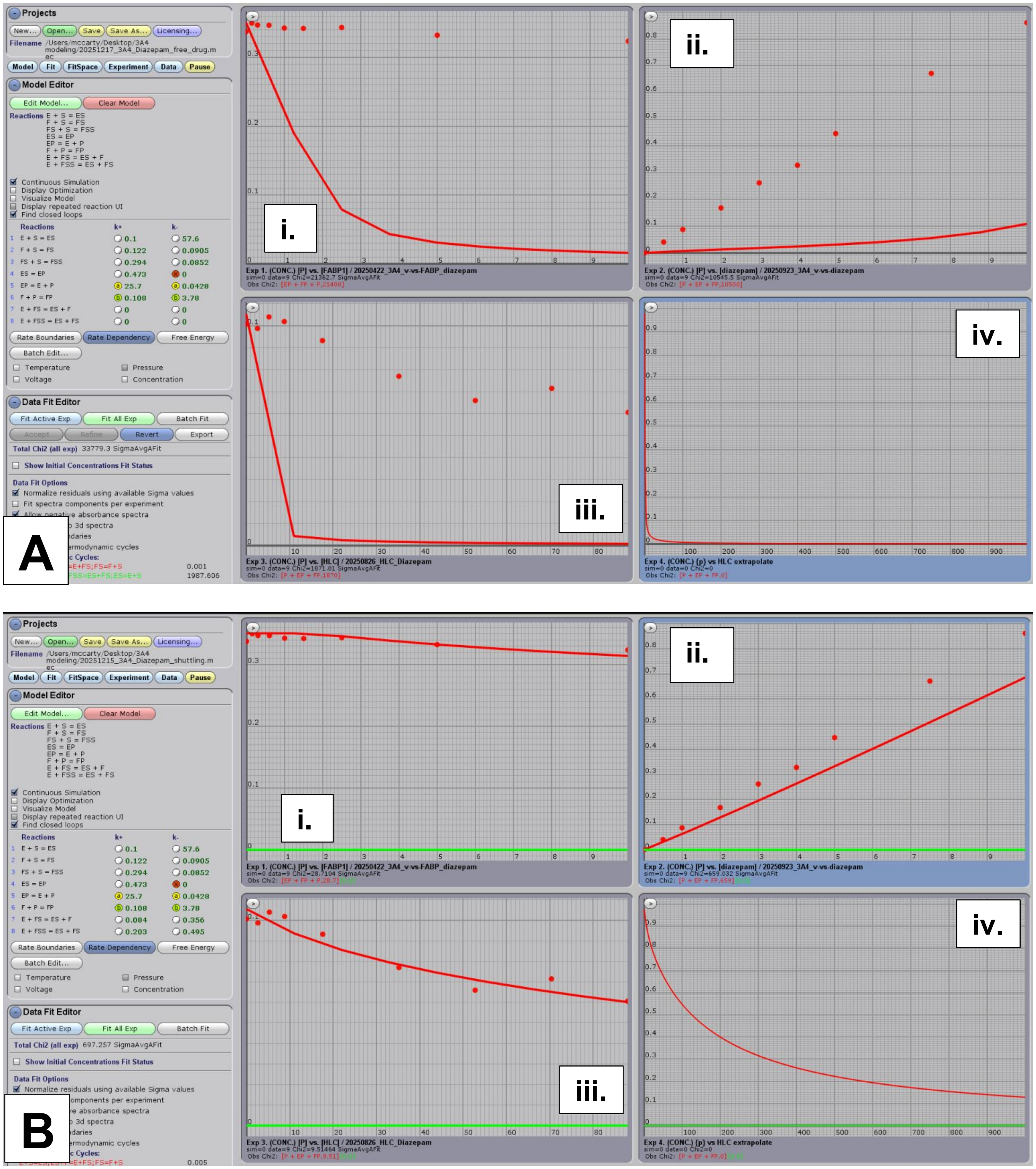

Figure S16. KinTek modeling of reactions of P450 3A4 with FABP1 and diazepam. Modeling of the free drug hypothesis (A) and the direct transfer hypothesis (B) for four experiments: i. [Product] vs. [FABP1] (Figure 6A); ii. [Product] vs. [Diazepam] (Figure 6B); iii. [Product] vs. [cytosolic FABP1] (Figure 8A), and extrapolated modeling of [Product] vs. [cytosolic FABP1] (Figure 8B). The kinetic model is displayed at left with each the value of each rate constant presented.

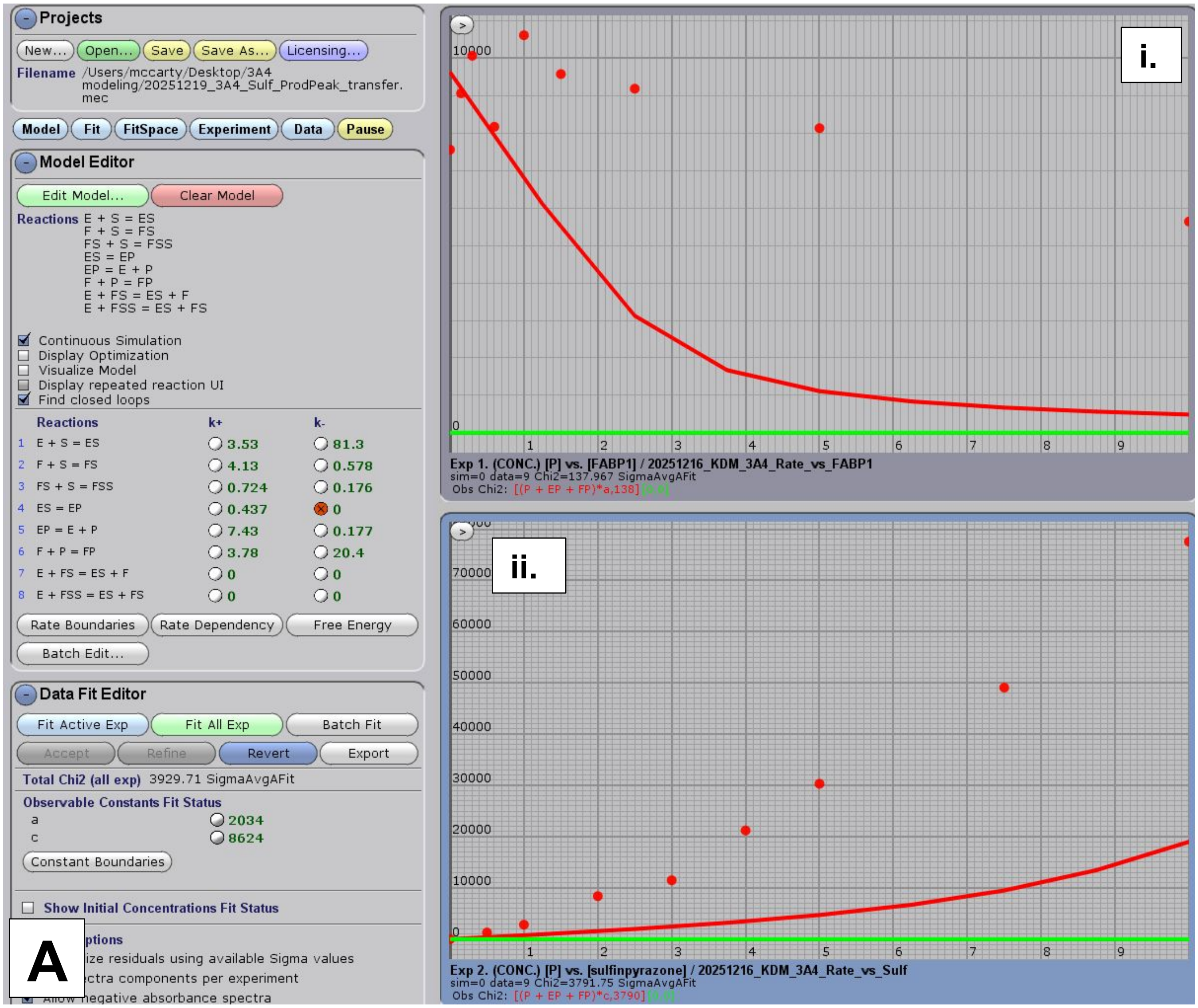
Projects
New... Open... Save Save As... Licensing...
Filename /Users/mccarty/Desktop/3A4 modeling/20251219_3A4_Sulf_ProdPeak_transfer.mec
Model Fit FitSpace Experiment Data Pause
Model Editor
Edit Model... Clear Model
Reactions E + S = ES
F + S = FS
FS + S = FSS
ES = EP
EP = E + P
F + P = FP
E + FS = ES + F
E + FSS = ES + FS
Continuous Simulation
Display Optimization
Visualize Model
Display repeated reaction UI
Find closed loops
Reactions k+ k-
1 E + S = ES 3.53 81.3
2 F + S = FS 4.13 0.578
3 FS + S = FSS 0.724 0.176
4 ES = EP 0.437 0
5 EP = E + P 7.43 0.177
6 F + P = FP 3.78 20.4
7 E + FS = ES + F 0 0
8 E + FSS = ES + FS 0 0
Rate Boundaries Rate Dependency Free Energy
Batch Edit...
Data Fit Editor
Fit Active Exp Fit All Exp Batch Fit
Accept Refine Revert Export
Total Chi2 (all exp) 3929.71 SigmaAvgAFit
Observable Constants Fit Status
a 2034
c 8624
Constant Boundaries
Show Initial Concentrations Fit Status
A
ptions
ize residuals using available Sigma values
ctra components per experiment
negative absorbance spectra
i.
10000
0
1 2 3 4 5 6 7 8 9
Exp 1. (CONC.) [P] vs. [FABP1] / 20251216_KDM_3A4_Rate_vs_FABP1
sim=0 data=9 Chi2=137.967 SigmaAvgAFit
Obs Chi2: [(P + EP + FP)*a,138]
ii.
70000
60000
50000
40000
30000
20000
10000
0
1 2 3 4 5 6 7 8 9
Exp 2. (CONC.) [P] vs. [sulfinpyrazone] / 20251216_KDM_3A4_Rate_vs_Sulf
sim=0 data=9 Chi2=3791.75 SigmaAvgAFit
Obs Chi2: [(P + EP + FP)*c,3790]

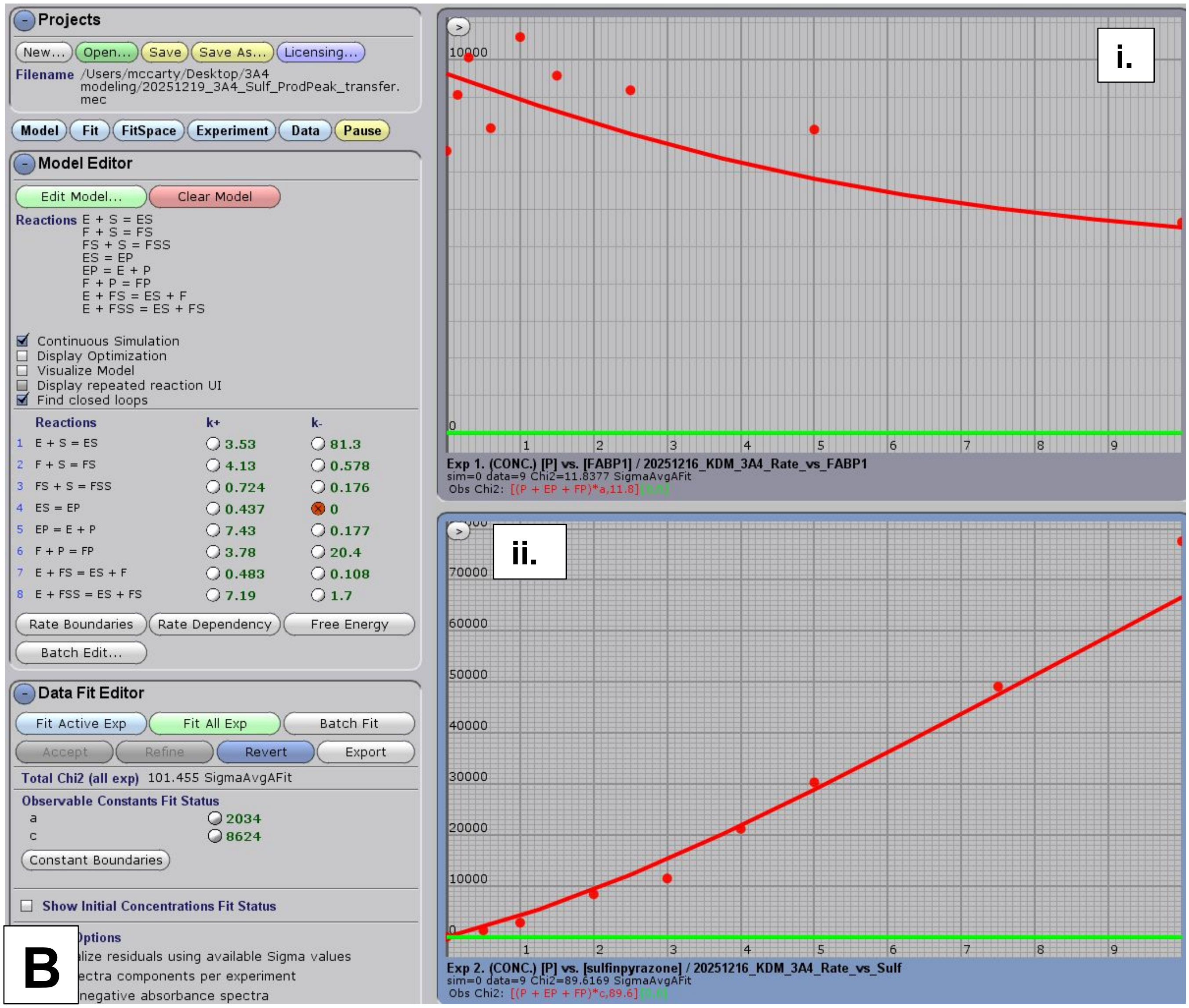

Figure S17. KinTek modeling of reactions of P450 3A4 with FABP1 and sulfinpyrazone. Modeling of the free drug hypothesis (A) and the direct transfer hypothesis (B) for two experiments: i. [Product] vs. [FABP1] (Figure 6C); ii. [product] vs. [sulfinpyrazone] (Figure 6D). The values of scalars (a, c) are input to convert peak area of the sulfone product (for which a standard was not available) to concentration.

References

(1) Laemmli, U. K. Cleavage of Structural Proteins during the Assembly of the Head of Bacteriophage T4. *Nature* **1970**, *227* (5259), 680–685. DOI: 10.1038/227680a0.
